# Natural bioactive compounds modified with mesenchymal stem cells: new hope for regenerative medicine

**DOI:** 10.3389/fbioe.2025.1446537

**Published:** 2025-05-09

**Authors:** Jingjing Wu, Ying Ge, Wendi Huang, Li Zhang, Juan Huang, Nanqu Huang, Yong Luo

**Affiliations:** ^1^ Department of Neurology, Third Affiliated Hospital of Zunyi Medical University (The First People’s Hospital of Zunyi), Zunyi, Guizhou, China; ^2^ Key Laboratory of Basic Pharmacology and Joint International Research Laboratory of Ethnomedicine of Ministry of Education, Zunyi Medical University, Zunyi, Guizhou, China; ^3^ National Drug Clinical Trial Institution, Third Affiliated Hospital of Zunyi Medical University (The First People’s Hospital of Zunyi), Zunyi, Guizhou, China; ^4^ Department of Gerontology, Third Affiliated Hospital of Zunyi Medical University (The First People’s Hospital of Zunyi), Zunyi, Guizhou, China

**Keywords:** mesenchymal stem cells, natural bioactive compounds, resveratrol, icariin, Ginkgo biloba extract, tanshinone IIA, astragaloside IV, curcumin

## Abstract

Mesenchymal stem cells (MSCs) have the potential to differentiate into various cell types, providing important sources of cells for the development of regenerative medicine. Although MSCs have various advantages, there are also various problems, such as the low survival rate of transplanted cells and poor migration and homing; therefore, determining how to reform MSCs to improve their utilization is particularly important. Although many natural bioactive compounds have shown great potential for improving MSCs, many mechanisms and pathways are involved; however, in the final analysis, natural bioactive compounds promoted MSC proliferation, migration and homing and promoted differentiation and antiaging. This article reviews the regulatory effects of natural bioactive compounds on MSCs to provide new ideas for the therapeutic effects of modified MSCs on diseases.

## 1 Introduction

There are many types of stem cells that can be divided into three categories: embryonic stem cells derived from early embryos, induced pluripotent stem cells, and adult stem cells, including haematopoietic stem cells, neural stem cells, and mesenchymal stem cells (MSCs) (Tian et al., 2023). MSCs were first discovered from bone marrow in 1976 (Lan et al., 2021) and have been found in almost all tissues in the human body. Compared with unipotent stem cells, stem cells have high self-renewal ability and good differentiation ability. The pluripotency of MSCs manifests in their ability to differentiate into bone cells, chondrocytes, fat cells, and other cell lines. Bone marrow and subcutaneous fat are the preferred sources of MSCs (Zhou and Shi, 2023; Eirin et al., 2024). MSCs can also be isolated from various adult tissues, such as adipose tissue (Gou et al., 2024), synovial membrane (Li et al., 2020), dental pulp (Yang et al., 2025), skin (Rendra et al., 2023), peripheral blood (Chen et al., 2023), nasal olfactory mucosa (Valipour et al., 2024), lung (Rangasamy et al., 2021), breast milk (Rahmani-Moghadam et al., 2022), muscle (Serteyn et al., 2024), periosteum (Ye et al., 2024), corneal limbus (Santra et al., 2024), endometrial and menstrual blood (Song et al., 2024), cervix (Eiro et al., 2024), and foetal/neonatal tissue (Thiruvenkataramani et al., 2024). MSCs are good candidates for future experimental or clinical applications and have received increasing attention (Matsuzaka and Yashiro, 2024; Pharoun et al., 2024; Shan et al., 2024). Stem cells have three important functions that make them potentially useful. The first is homing, which is the chemotactic effect of stem cells when damage occurs in the body (Han et al., 2022). The second is differentiation, whereby stem cells can differentiate into a variety of cell types by means of transplantation and by adding or replacing damaged tissue to enhance functional recovery (Nie et al., 2023). The third is the secretion of bioactive factors, which may influence local and systemic physiological processes (Daneshmandi et al., 2020). The most important function of stem cells is self-renewal (Wilkinson et al., 2020).

**FIGURE 1:**
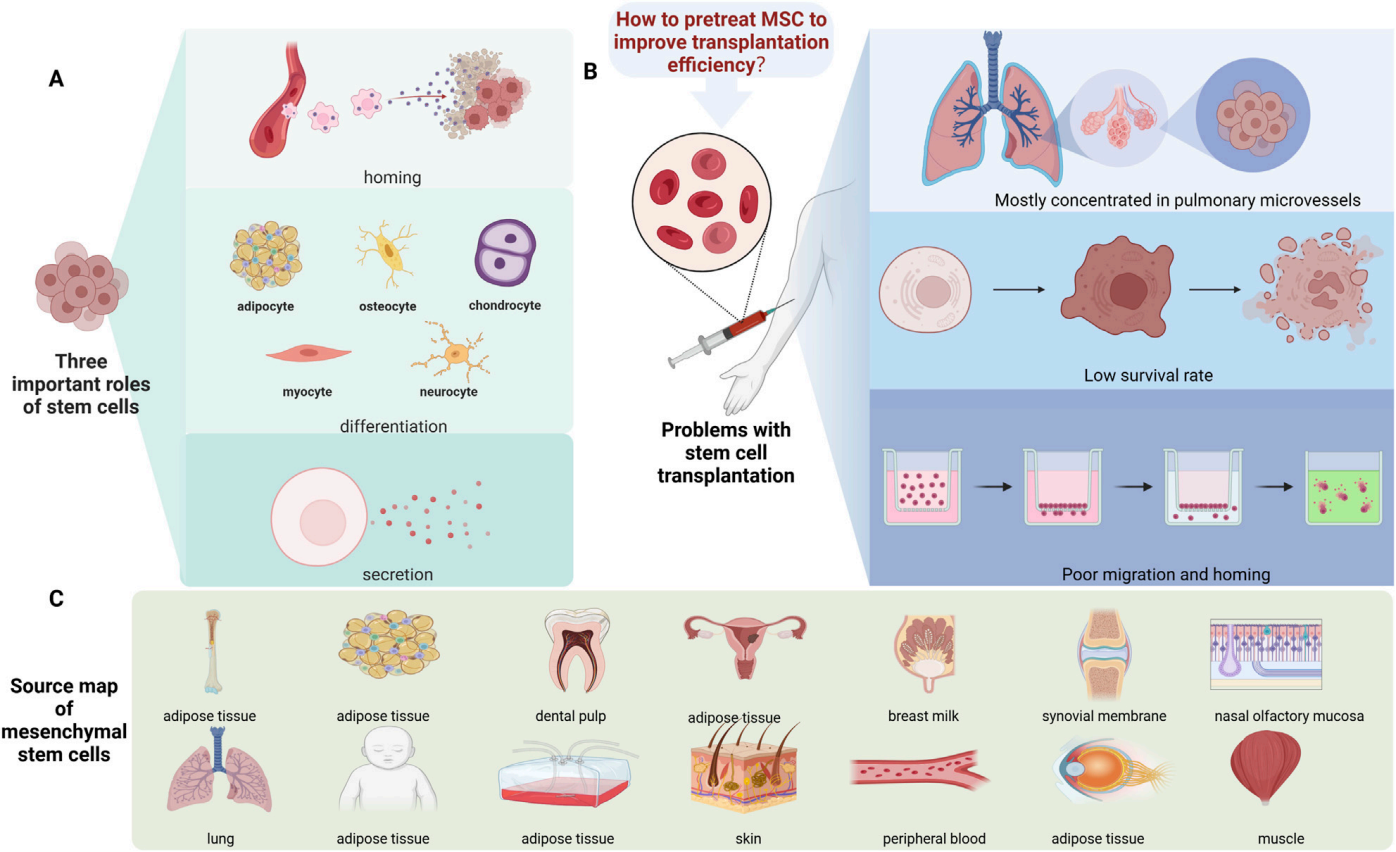
Source and function summary diagram of the MSCs. **(A)** Three important functions of stem cells: homing, differentiation, and secretion. **(B)** The main challenges associated with stem cells are their higher concentration in pulmonary microvessels, low survival rates, and poor migration and homing. **(C)** The source of the stem cells.

The contribution of stem cells to modern medicine is crucial. Not only because they can be widely used in basic research but also because they offer more and better opportunities to develop new treatment strategies (Pharoun et al., 2024). Their properties make them valuable for a wide range of applications in the biological and medical sciences (Lu et al., 2025). MSCs play a wide range of physiological roles, including maintaining tissue homeostasis and regeneration (Zhidu et al., 2024), as well as modulating immune effects (Hazrati et al., 2024). As a result, indications for MSCs have expanded to include graft-versus-host disease, multiple sclerosis, Crohn’s disease, amyotrophic lateral sclerosis, myocardial infarction, and acute respiratory distress syndrome (ARDS) (Han et al., 2022; Pharoun et al., 2024). Over the past decade, several preclinical studies and more than 5,000 clinical trials involving MSCs have been registered, with more than 1,500 completed (Al-Azab et al., 2023). In addition, more than 100 clinical trials have tested stem cells for regenerative medicine, and MSCs have great potential in treating Parkinson’s disease, among other conditions (Abbott, 2025). Although the clinical application of stem cells is promising, there are still some challenges that warrant consideration in cell therapy and regenerative medicine. Studies have shown that most MSCs lose their biological activity within a week, even after the orthotopic transplantation of MSCs (Preda et al., 2021). Moreover, most transplanted MSCs are concentrated in pulmonary microvessels, and the amount of targeted tissues is limited (Shan et al., 2024). Therefore, even though MSCs have various advantages, there are also various challenges, such as the low survival rate of transplanted cells and poor migration and homing of MSCs; thus, the function of MSCs is limited. Currently, various studies have shown that they can increase the survival, migration, proliferation and differentiation of stem cells; promote homing; and induce the release of nutrient factors in various ways to further promote the recovery of neural function (Han et al., 2024). Some studies have been conducted to pretreat stem cells before injection to change some of the characteristics of the cells, thereby improving the efficacy of stem cell transplantation (Huang et al., 2018; Yu et al., 2023) (Figure 1).

According to the World Health Organization (WHO), two-thirds of the world’s population has access to herbal medicines. Therefore, the WHO encourages the inclusion of plants in treatment regimens for various diseases to improve their effectiveness and reduce potential costs (Mekhemar et al., 2020). Natural bioactive compounds are defined as chemical substances derived from natural sources (such as plants, animals, or microorganisms) that have biological effects on living organisms, tissues, or cells. Plants constitute one of their main sources (Li et al., 2024). Since ancient times, plant-derived natural compounds have been widely used to treat various diseases (Gugliandolo et al., 2020; Huang et al., 2024). In recent years, increasing attention has been given to natural bioactive compounds, and the modification of stem cells is a new research direction. They are able to modulate stem cell self-renewal and differentiation potential and target a wide range of types of intracellular signal transduction (Feng et al., 2022; Li et al., 2023; Li et al., 2024). An increasing amount of data show that natural bioactive compounds may have different protective mechanisms through their antioxidants, free radical scavengers, harmful metal ion chelators, regulation of cell survival genes and signals, and antiapoptotic activity, so they have a wide range of applications in the treatment of many diseases (Huang et al., 2023; Pacyga et al., 2024; Wang et al., 2024). In addition to treating diseases, some natural bioactive compounds can also play an antioxidant role by activating the proliferation of lymphocytes to enhance immune function, increasing the number of natural killer (NK) cells and scavenging free radicals (Kim et al., 2022; Mahmoudi et al., 2024). Studies have shown that natural bioactive compounds may be able to effectively regulate the proliferation and differentiation of stem cells (Lakshminarayanan et al., 2025). Therefore, natural bioactive compounds have the potential to be a new source for improving stem cells, increasing their proliferation and improving their function. In this work, the regulatory effects of natural bioactive compounds on MSCs were reviewed to provide better theoretical support for the improvement of stem cells and enhance their application in clinical and basic research.

## 2 Overview of MSCs

MSCs are pluripotent stem cells that can self-renew and differentiate into multilineage cells (Lin et al., 2022). These cells have the ability to differentiate into osteoblasts, chondrocytes, and adipocytes, and the cell phenotypes CD73, CD90, and CD105 are positive, while the major histocompatibility complex class II (MHC-II), CD11b, CD14, CD31, CD34, and CD45 are negative (Zhou and Shi, 2023; Cao et al., 2024). The quality and criteria of MSCs should include cell surface markers, differentiation potential and other necessary parameters. These parameters include cell surface labelling profiles, bone formation capacity in the model, as well as cell and particle sizes, telomere length, state of ageing, secretion of trophic factors (secretomes), and immune regulation (Trivanović et al., 2015; Samsonraj et al., 2017). Because MSCs are easy to extract from foetal and adult tissues and do not require overly demanding cell culture conditions, they are promising research targets. Many studies have confirmed their anti-inflammatory components, multilineage potential, tissue regeneration ability and immunomodulatory effects, so they have been widely studied in the treatment of many diseases (Al-Azab et al., 2022; Gopalarethinam et al., 2023).

### 2.1 Bone marrow mesenchymal stem cells (BMSCs)

BMSCs are the most widely used stem cells in regenerative medicine and tissue engineering. They are nonhematopoietic stem cells that exist in the bone marrow and have pluripotent differentiation potential (Zhang et al., 2023). However, the mobilization of BMSCs from the bone marrow and their migration to damaged tissue during healing are key issues. The migration of BMSCs to target tissues is a complex biological process. This transport process is affected by many factors, such as chemical factors (e.g., chemokines, cytokines, and growth factors) and mechanical factors (e.g., shear stress and vascular circulation) (Fu et al., 2019). At present, several methods are used to evaluate the migration of BMSCs, the most common methods are the Transwell method and the scratch test (Sun and Fan, 2025). Moreover, BMSCs can also be labelled with fluorescence to track their location (Yun et al., 2023). The therapeutic use of BMSCs also has certain limitations, such as cell rejection, poor immune response, toxicity, tumorigenicity, potential contamination by viruses and problems with cell transport and storage prior to transplantation (Tayebi et al., 2022; Zhang et al., 2022).

### 2.2 Human umbilical cord mesenchymal stem cells (HUCMSCs)

The human umbilical cord is a promising source of mesenchymal stem cells (HUCMSCs). Unlike that for BMSCs, the process of HUCMSC collection is painless, and HUCMSCs exhibit faster self-renewal. HUCMSCs are a better source than commonly used embryonic stem cells. They can differentiate into three types of germ layers that promote tissue repair, regulate the immune response, and have anticancer properties (Rodríguez-Eguren et al., 2022). However, HUCMSCs also have certain limitations, such as maintaining biological activity and quantifying bioactive substances (Ding et al., 2018).

### 2.3 Adipose-derived mesenchymal stem cells (ASCs)

ASCs are generally isolated from heterogeneous cell populations produced in the stromal vascular fraction (SVF) of adipose tissue. The SVF is obtained from subcutaneous fat tissue from individuals who undergo surgical procedures such as liposuction to remove excess, unwanted fat, so easy access to sources is one reason for its widespread use (Yaylacı et al., 2023). ASCs can self-renew and differentiate into multiple cell lines. ASCs have been proven to have anti-inflammatory, antifibrotic, antiapoptotic and proangiogenic effects both *in vitro* and *in vivo.* Therefore, they have been widely used in cell therapy and regenerative medicine (Al-Ghadban et al., 2022; Zeng et al., 2022; Barone et al., 2023). ASCs are widely used in many diseases, such as multiple sclerosis, diabetes, Crohn’s disease, SLE, and graft-versus-host disease (Al-Ghadban et al., 2022). Unlike BMSCs, ASCs differ in that their fatty acid translocase marker CD36 and cell adhesion marker CD106 are not expressed (Poon et al., 2020). Previous reports have shed new light on the ability of ASCs to differentiate. ASCs can differentiate into various types of epithelial cells, such as renal tubular epithelial cells and retinal pigment epithelial cells (Cawthorn et al., 2012). However, other researchers have demonstrated that these cells may have less potential for bone formation and cartilage formation than BMSCs. The importance of adipose tissue-derived stem cells has been questioned (Al-Sammarraie et al., 2024).

### 2.4 Other types of mesenchymal stem cells

Other well-known sources of adult MSCs include the cervix, placenta, amniotic fluid, dental pulp, breast milk, and synovial membrane. Compared with other methods used to obtain other MSCs (such as bone marrow or adipose tissue), human cervical stem cells (hUCESCs) are also a type of MSC that can be obtained via cervical smears, are easy to separate, have a high proliferation rate, and can yield a greater number of hUCESCs or exosomes and other derivatives, which is conducive to basic research and clinical applications (Vizoso et al., 2017; Ge et al., 2024). Compared with MSCs from other sources, human placental mesenchymal stem cells (hPMSCs) have the advantages of stable proliferation and low immunogenicity. These properties make hPMSCs ideal materials for stimulating tissue repair (Lu et al., 2021; Yu et al., 2024). Amniotic fluid MSCs are a type of MSC produced during the perinatal period. Compared with adult MSCs such as BMSCs, their advantages include a minimally invasive separation process, more primitive cell characteristics, nontumorigenicity, and low immunogenicity. Currently, they have received increasing attention (Doktor et al., 2025). On the basis of the clinical diagnosis of nonfunctional or pathogenic tissues in the mouth, oral MSCs can be obtained through minimally invasive surgery. In addition, they are often discarded as medical waste after they are removed from the mouth. Therefore, compared with the use of MSCs from other tissues, this is a unique advantage of dental MSCs in research (Masuda et al., 2021).

## 3 Effects of different types of natural bioactive compounds on MSCs

**FIGURE 2:**
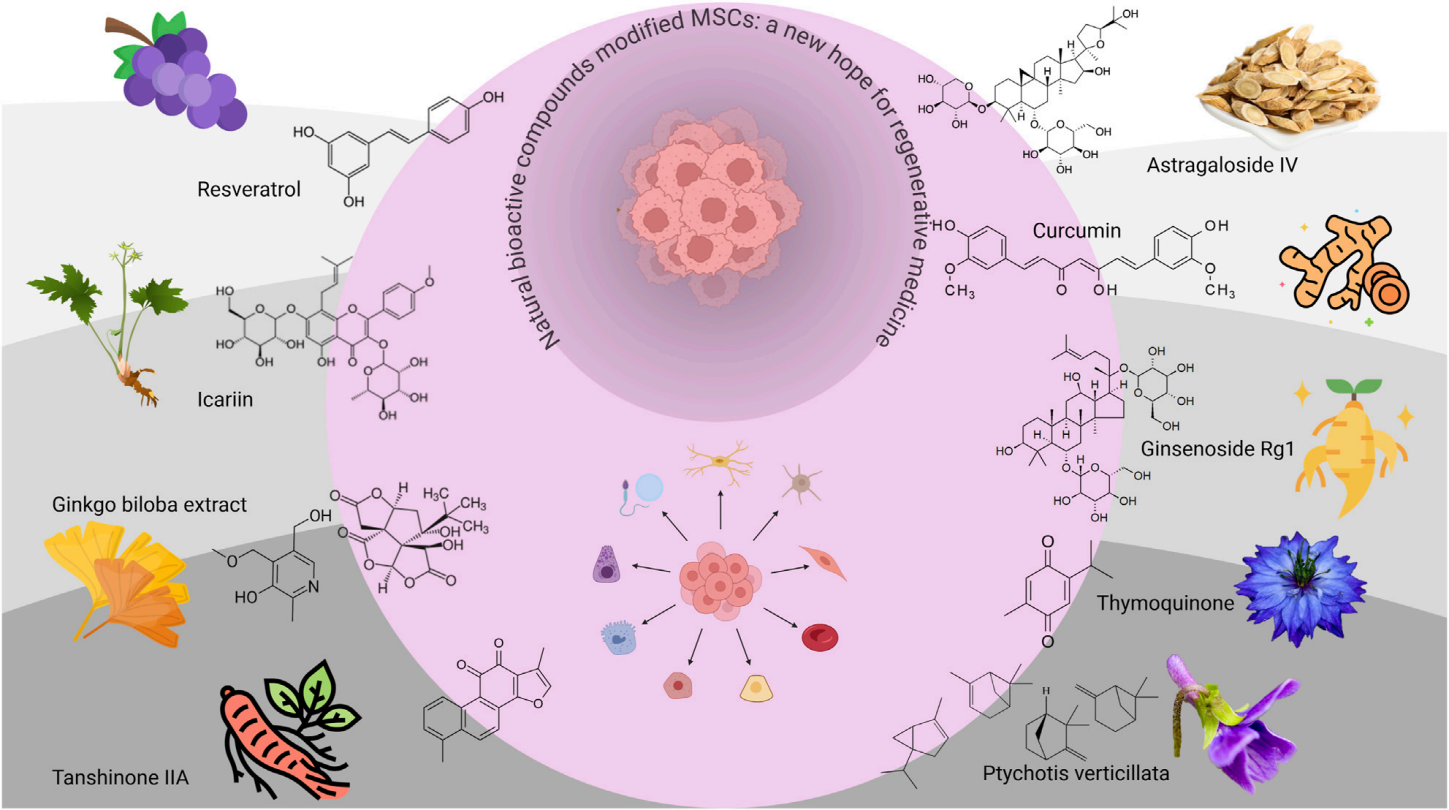
Effects of different types of natural bioactive compounds on MSCs. This simple schematic diagram mainly illustrates the effects of different types of natural bioactive compounds on MSCs. The chemical structures and main sources of the nine representative ingredients on the grey outside the circle include resveratrol (RSVL), icariin (ICA), Ginkgo biloba extract (GBE), tanshinone IIA (Tan IIA), astragaloside IV (AGS-IV), curcumin, ginsenoside Rg1, thymoquinone (TQ), and Ptychotis verticillata (PV).

Because MSCs are good candidates for clinical and basic research, it is especially important to obtain adequate numbers of these cells. They are usually cultured *in vitro* with animal serum and various growth factors. However, repeated freeze‒thaw cycles, *in vitro* culture conditions and continuous passages during the culture process have adverse effects on the proliferation of MSCs, such as reduced self-renewal, increased cell senescence, increased apoptosis and premature differentiation, and these cells are very susceptible to the influence of the microenvironment (Yang et al., 2018; Preda et al., 2021). A variety of natural bioactive compounds are used worldwide to treat and prevent various diseases, and they have multitarget, multilevel and multipathway characteristics (Huang et al., 2023). The bioactive compounds naturally present in seaweed, herbs, fruits, and vegetables have the ability to regulate the self-renewal and differentiation potential of adult stem cells. They mainly target a wide range of intracellular signal transduction pathways (Li et al., 2022). Natural plant compounds can improve the rate of tissue regeneration, which has certain advantages in the tissue engineering of stem cell therapy and alternative therapy. Although bioactive ingredients have not been widely used in the clinic because of their variability and complexity, a growing number of studies have focused on the modified effects of natural compounds on MSCs (Table 1) (Figure 2).

**TABLE 1:** Effect of natural bioactive compounds modified mesenchymal stem cells.

Compounds	Sources of MSC	Function	Mechanism of action	Ref
Icariin	BMSC	1.Promote proliferation and osteogenic differentiation of BMSC, inhibit lipogenic differentiation	1.Upregulated Runx2, ALP and I collagen promote bone formation, the expression of PPARγ, adipsin, CCAAT/C/EBPα and FABP4 mRNA was inhibited to inhibit the differentiation of BMMSC into adipocytes	Sheng et al. (2013), Huang et al. (2017), Liu et al. (2017), Wu et al. (2017), Lim et al. (2018)
			2.Activation of STAT-3 increases the activity and expression of cysteine (C)-X-C motif chemokine receptor 4 (CXCR4)	
		2.Promote migration of BMSCs	3. Stimulate the MAPK signaling pathway	
			4.Stimulation of Wnt/β-catenin signaling pathway	
	ASCs	Promote osteogenic differentiation	The mRNA expression of ALP, Col-1 and OC, Dlx5 and Runx2, BMP-2, -4 and -7 genes was stimulated. Improve Runx2, bmp and OC levels	Yang et al. (2019), Sharifi et al. (2020)
Resveratrol	BMSC	Promote proliferation and osteogenic differentiation of BMSC, inhibit lipogenic differentiation. Promote homing of MSC.	Runx2 gene expression on the SIRT1/FOXO3A axis can also be upregulated through ERK1/2 and MAPK signaling pathways	Dai et al. (2007), Tseng et al. (2011), Okay et al. (2015)
	HUCMSCs	Increase cell vitality, slow down aging and aging	Increase SIRT1 level, inhibiting the expression of p53 and p16	Wang et al. (2016)
	ASCs	Enhance the epithelial and osteogenic differentiation of MSCs	ASC differentiation was induced on collagen scaffolds	Wang et al. (2018)
Ginkgo biloba extract	BMSC	Promote proliferation and osteogenic differentiation of BMSC	1.Promotes osteogenesis by up-regulating BMP and Wnt/β-catenin signaling pathways	Gu et al. (2015)
			2. Upregulated PAX6 expression in cells	
Tanshinone IIA	BMSC	1.Better anti-neuroinflammatory effects	1.Downregulated BACE1 expression	Zhang et al. (2018), Huang et al. (2019), Yuan et al. (2022)
		2.Promote the differentiation of neuron cells	2.It may improve the survival rate of transplanted BMSCs, increase the level of nutrient factors secreted by BMSCs to the lesion area, and reduce inflammatory cytokines	
		Promote hBMSCs proliferation	3.Increase the release of fibroblast growth factor 2 (FGF2)	
	Human periodontal ligament stem cells (hPDLSC)	It promotes this differentiation and hPDLSC maturation	Osteogenesis of hPDLSC was induced by the ERK1/2-Runx2 axis	Liu et al. (2019)
	MSC	Regulate the migration ability of MSC.	Regulate the expression of CXCR4	Xie et al. (2013)
Curcumin	BMSC	1.Delay the aging	1.Activated autophagy	Gu et al. (2012), Wang et al. (2019), Chen et al. (2021), Deng et al. (2021)
			2.It is associated with HO-1 expression	
		2.Promote osteogenic differentiation. Inhibition of lipogenic differentiation	The expression of Kruppel-like factor 15 was inhibited	
	ASCs	Delay the aging	1.Increase the expression of TERT gene	Liu et al. (2015), Pirmoradi et al. (2018)
			2.Increased tolerance to oxidative stress damage	
	hDP-MSC	Enhance immune regulation and regeneration		Ayadilord et al. (2022)
Astragaloside	MSC	Promotes angiogenesis of endothelium-like cells	Upregulated expression of Cx37, Cx 40 and Cx43	Li et al. (2018)
	ASCs	1.Enhance its ability to proliferate	1.Decreased PC-I secretion and increased MMP-1 release were induced in fibroblasts	Niu et al. (2020), Wang et al. (2022)
		2.Promote migration and homing	2.FAK phosphorylation induced by CXCR2	
	hBMSCs	Ability to promote proliferation and differentiation	Regulated by the miR-124-3p.1/STAT3 axis	Cao et al. (2021)
Ginsenoside Rg1	BMSCs	1.Delay the aging	1.Inhibition of phosphorylation of GSK-3β	Gu et al. (2016), Wang et al. (2020), Wang et al. (2021)
			2.Activation of AKT or NRF2	
		2.Promote osteogenic differentiation	3.Example Activate the GR/BMP-2 signal path	
Thymoquinone	G-MSCs	Promotes cell transformation into an immunocompetent differentiated phenotype	Increase the expression of TLR3	Mekhemar et al. (2022)
	Breast CSCs	Wnt/PI3K signaling pathway	Altered angiogenesis capacity and mesenchymal to epithelial transformation	(Haiaty et al., 2021b; a)
	MSCs	Promote differentiation	Triggers inhibition of NF-κB signaling	Rahmani-Moghadam et al. (2021), Banu (2022), Ishaque et al. (2022)
Ptychotis verticillate		1. Promote migration	PI3K and MAPK signaling pathways	Maeda, 2020; Najar et al. (2024)
		2. Immunomodulation		

### 3.1 Resveratrol

Resveratrol (RSVL) is a natural polyphenol plant antitoxin (Huang et al., 2020; Huang et al., 2023) and is a natural polyphenolic phytoestrogen. It was first found in the root extract of white mustard (*Veratrum grandiflorum*) and was found in the roots of *knotweed* in 1963 (Vauzour, 2012). RSVL has two configurations, cis-type or trans-type structures, among which trans-RSVL is the main form existing in nature and the most widely studied form (Huang et al., 2020). RSVL has a wide range of biological activities, including antioxidant, antiviral, anti-inflammatory, antiaging and anticancer properties (Khattar et al., 2022). It has great potential to prevent a variety of acute and chronic diseases. Recent studies have shown that it can play a reliable rejuvenating role in various animal models, tissues and organs, especially in the modification of stem cells. Studies have shown that RSVL can improve the therapeutic effect of MSCs by increasing their survival rate, self-renewal ability and antiaging effects (Hu and Li, 2019). RSVL is also involved in the regulation of bone formation and fat, as well as nerve regeneration, in MSCs (Joe et al., 2015).

In terms of the antiaging effects of MSCs on BMSCs, studies have shown that RSVL can activate Sirtuin 1 (SIRT1), reduce β-catenin activity, increase extracellular signal-regulated kinase (ERK) phosphorylation and glycogen synthase kinase-3 beta (GSK-3β) phosphorylation, improve the self-renewal potential of early-passage MSCs and improve their pluripotency (Yoon et al., 2015). Different concentrations of RSVL had different antiaging effects on HUCMSCs. At 0.1, 1 and 2.5 μM, RSVL promoted cell viability and slowed the ageing of MSCs by increasing SIRT1 expression and inhibiting p53 and p16 expression. At concentrations of 5 and 10 μM, RSVL can increase the degree of senescence and apoptosis of MSCs by inhibiting SIRT1 and PCNA and stimulating the expression of p53 and p16 (Wang et al., 2016). Therefore, not all concentrations of RSVL are beneficial for the growth of MSCs.

RSVL is involved in the differentiation of MSCs. Studies have shown that RSVL can enhance the osteogenic differentiation ability of BMSCs by increasing NO production and the cGMP content, activating ERK1/2 and p38 mitogen-activated protein kinase (MAPK), and upregulating the expression of mitogens (Song et al., 2006; Dai et al., 2007; Lv et al., 2018). RSVL can also replace insulin in lipid-forming medium, increase the phosphorylation of cyclic AMP response element binding protein (CREB), and induce lipid differentiation in MSCs (Caldarelli et al., 2015).

RSVL has also been shown to improve the regulation of nerve regeneration. In BMSCs, RSVL can upregulate AMPK/SIRT1 signal transduction, thereby increasing the level of neural progenitor cell markers in MSCs isolated from ALS patients (Yun et al., 2019). In dental pulp stem cells, RSVL can increase the expression of the neuron-specific marker genes *Nestin*, *Musashi* and *NF-M*, thereby promoting the differentiation of dental pulp stem cells into neuronal cells (Geng et al., 2017). RSVL can activate the phosphatidylinositol 3-kinase (PI3K) signalling pathway or increase the levels of protein kinase A, GSK-3β, and ERK1/2 in HUCMSCs, thereby increasing the expression of neural markers and promoting neural repair (Jahan et al., 2018a; Jahan et al., 2018b). Moreover, RSVL can play a positive role in the treatment of periodontitis by promoting the proliferation, osteogenic differentiation and immune regulation of MSCs (Jiang et al., 2023).

Thus, although RSVL can regulate the survival of MSCs, it can promote their self-renewal and maintain their pluripotency. However, to some extent, the concentration of RSVL, the time of administration and the method and time of pretreatment all affect the effect of RSVL on MSCs. Moreover, although many studies have focused on RSVL, few studies have investigated RSVL derivatives. Perhaps related studies can be conducted in the future, which will contribute to further exploration of the improvement effect of RSVL on MSCs in regenerative medicine. There are also several contradictory conclusions in the research. For example, some studies have shown that RSVL can help MSCs maintain pluripotency (Yoon et al., 2015), but other studies have shown that RSVL can lead to the production of neural markers in HUCMSCs (Jahan et al., 2018a; Jahan et al., 2018b). This may be related to the different types of MSCs, doses and intervention methods used.

### 3.2 Icariin (ICA)

ICA is the main bioactive compound of *Epimedium brevicornum Maxim* (Hsieh et al., 2011). The composition of ICA includes a C-3 glucose group, a C-4 methoxy group, a C-8 isoprene group and a C-7 rhamnosyl group, and it is a class of isoprene flavonoid compounds. The molecular formula of ICA is C_33_H_40_O_15,_ and its molecular weight is 676.67 g/mol (Sharifi et al., 2020). ICA has a variety of pharmacological activities, including hormone-like, antitumour, immunomodulatory and antioxidant effects (Bi et al., 2022; Seyedi et al., 2023). ICA has been used to treat many diseases, such as preventing osteoporosis, improving sexual dysfunction, regulating immune system function and improving cardiovascular function (He et al., 2020).

As mentioned earlier, while the use of MSCs has many advantages, many methods have been developed, which limits their application. Therefore, new drugs have been developed to promote the proliferation and differentiation of MSCs. The effects of ICA have been extensively studied in animal and *in vitro* models, and its ability to modify MSCs has attracted much attention. The beneficial effect of ICA on MSCs involves promoting osteogenic differentiation and inhibiting lipogenic differentiation. Some studies suggest that this effect is achieved by inactivating GSK-3β and inhibiting the expression of PPARγ (Sheng et al., 2013). Some studies suggest that this effect is related to the BMP (BMP-2, BMP-4, BMP-7) and MAPK/ERK pathways (Wu et al., 2017). Some studies suggest that it promotes osteogenesis through the upregulation of Runx2, ALP and I collagen and the Notch signalling pathway and inhibits *PPARγ*, *adipsin* gene expression, and CCAAT/enhancer binding protein α (*C/EBPα*) and fatty acid binding protein 4 (*FABP4*) mRNAs (Huang et al., 2017; Liu et al., 2017). EGb 761 may be associated with findings in other studies that have shown that ICA can activate STAT-3 and increase the activity and expression of cysteine (C)-X-C motif chemokine receptor 4 (CXCR4) (Lim et al., 2018), thus promoting the proliferation and osteogenic differentiation of MSCs. Moreover, ICA can promote BMSC migration by stimulating the MAPK signalling pathway (Jiao et al., 2018).

In addition to BMSCs, hADSCs can effectively promote osteogenic differentiation by stimulating the mRNA expression of bone matrix protein (*ALP*, *Col-1*, and *OC*), bone transcription factor (*Dlx5* and *Runx2*), and bone morphogenetic protein (*BMP-2*, *-4*, and *-7*) genes (Yang et al., 2019; Sharifi et al., 2020). The beneficial effect of ICA on MSCs involves mainly promoting osteogenic differentiation and inhibiting lipogenic differentiation, and studies on whether ICA can promote proliferation, survival, homing and migration are limited, which also provides ideas for future studies.

### 3.3 Ginkgo biloba extract (GBE)

GBE is a bioactive component of *Ginkgo biloba L.* with various pharmacological activities (Hu et al., 2023). Its two main extracts have similar compositions: EGb 761, which has 24% ginkgo flavonoid glycosides and 6% terpenoids, and LI1370, which is composed of 25% ginkgo flavonoid glycosides and 6% terpenoids. GBE has been widely used to treat cerebrovascular insufficiency, peripheral vascular insufficiency, and cognitive impairment associated with ageing and neurodegenerative diseases (such as Alzheimer’s disease) because of its ability to improve high blood pressure, antithrombosis, inflammation, oxidative stress, and infection (Mousavi et al., 2022; Xie et al., 2022). Specifically, GBE can reduce oxidative stress and inhibit the expression of inflammatory cytokines. In addition, studies have confirmed that GBE can promote the growth and proliferation of different cell types, such as neural stem cells, endothelial progenitor cells, and cochlear hair cells (Gu et al., 2015).

Studies have reported that GBE can promote the growth and differentiation of a variety of cells, including neural stem cells and endothelial progenitor cells (Zheng et al., 2018). Studies have shown that when GBE is in the range of 25–75 mg/L, GBE can promote the proliferation and osteogenesis of human BMSCs by upregulating the BMP and Wnt/β-catenin signalling pathways. When the concentration of GBE was 100 mg/L or above, the ability of GBE to promote the proliferation of BMSCs weakened or disappeared (Gu et al., 2015). The concentration of GBE is particularly important for the modification of BMSCs, which directly determines whether the growth of MSCs is promoted or inhibited; thus, special attention should be given to GBE concentrations in future studies. In rat models of myocardial infarction, EGb 761 may mediate the protective effect of EGb on myocardial infarction by increasing the implantation rate of MSCs, thereby improving the viability and differentiation of cardiomyocytes (Liu et al., 2014). Other studies have shown that MSCs modified with GBE may have good applications in the treatment of local inflammation and the oxidative microenvironment (Gu et al., 2015). Hence, these findings underscore the concentration-dependent therapeutic potential of GBE in stem cell-based neuroregeneration.

### 3.4 Tanshinone IIA (Tan IIA)

Tan IIA, a bioactive ingredient isolated from the herb *Salvia miltiorrhiza Bge*, is an antioxidant and anti-inflammatory agent that inhibits the cytotoxicity of damaged tissues, significantly improves blood circulation and delays tumour progression (Zeng et al., 2021; Ng et al., 2022). Studies have shown that Tan IIA has the potential to reduce oxidative stress by regulating the levels of antioxidant enzymes, including glutathione peroxidase (GPx), superoxide dismutase (SOD), and catalase. The anti-inflammatory effect of Tan IIA may be mediated by weakened inflammatory mediators in RAW264.7 macrophages, namely, interleukin (IL)-1β, IL-6 and tumour necrosis factor (TNF)-α (Ansari et al., 2021).

With respect to the modification effect of Tan IIA on MSCs, Tan IIA can regulate the migration ability of MSCs. Studies have shown that Tan IIA is at least partially regulated by regulating CXCR4 expression (Xie et al., 2013). Tan IIA can improve the survival rate of MSCs, and some researchers have reported that Tan IIA can promote the differentiation of transplanted BMSCs into neurocell-like cells in SCI models. The hypothesized mechanism is that TIIA can improve the survival rate of transplanted BMSCs, increase the level of nutrient factors secreted by BMSCs to the lesion area, and reduce the secretion of inflammatory cytokines (Zhang et al., 2018). Tan IIA may better induce the osteogenic ability of MSCs. Studies have confirmed that Tan IIA can induce hPDLSC osteogenesis through the ERK1/2-Runx2 axis, which provides a choice in regenerative medicine approaches for the treatment of periodontitis (Liu et al., 2019). However, Tan IIA-modified MSCs can play a better role; for example, researchers have reported that Tan IIA-pretreated stem cells can reduce neuronal death by increasing the levels of anti-inflammatory cytokines (such as IL-4, -6, -8, and -13) and reducing inflammation through the PI3K/Akt/mTOR and/or TREM2 signalling pathways (Dai et al., 2017; Huang et al., 2022; Kaiser et al., 2022; Wu et al., 2024). Studies have also shown that TIIA-MSCs can significantly improve the learning and memory ability of Aβ_25-35_ model rats and are more effective than MSCs. The protective mechanism may involve promoting the survival of hippocampal neurons by downregulating BACE1 expression and regulating neuroinflammation-related cytokines (Huang et al., 2019). In general, many studies have investigated the modification effect of Tan IIA on MSCs, but the specific mechanism of action of Tan IIA on MSC modification still needs further study.

### 3.5 Astragaloside IV (AGS-IV)

AGS-IV is one of the main compounds of *Astragalus membranaceus* water extract and is a cycloartemisane-type triterpenoid glycoside chemical. Several studies have shown that AGS-IV has a strong protective effect on cardiovascular, lung, kidney, and brain-related diseases (Zhang et al., 2020). The pharmacological effects of AGS-IV are multifactorial and include anti-inflammatory effects via the inhibition of inflammatory factors, increased proliferation of T and B lymphocytes, and the inhibition of neutrophil adhesion-related molecules. Moreover, it has neuroprotective, antifibrotic and antitumour effects (Liang et al., 2023).

The ability of AGS-IV to modify MSCs is first reflected in the increased proliferative and paracrine activities of these cells. AGS-IV-treated ADSCs can significantly reverse the UV-B-induced decrease in PC-I secretion and increase MMP-1 release in fibroblasts. In addition, ASI-treated ADSCs significantly increased dermal thickness, collagen content, and microvascular density in the photoaged skin of nude mice (Niu et al., 2020). Second, AGS-IV has a positive effect on the cell cycle and osteogenic differentiation of MSCs. Studies have shown that AGS-IV can promote the activity of hBMSCs, the cell cycle, ALP activity and osteogenic differentiation through the miR-124-3p.1/STAT3 axis while increasing the expression of osteoblast marker molecules (Cao et al., 2021). Furthermore, AS-IV improved ADSC migration, angiogenesis, and endothelial recruitment. *In vivo*, AS-IV-pretreated ADSCs have greater angiogenesis potential and better therapeutic efficacy in ischaemic hindlimb models. The molecular mechanism may be related to the upregulation of CXCR2 to promote the phosphorylation of FAK (Wang et al., 2022). In contrast to other biological compounds, some studies have combined the modification effect of AGS-IV on MSCs with other biological compounds to explore the modification effect on MSCs. Studies have shown that the combination of tanshinone IIA and astragaloside IV can promote the proliferation and differentiation of MSCs. The mechanism may involve regulating the mobilization of MSCs by regulating the expression of CXCR4. Similarly, studies have shown that they can promote angiogenesis of endothelium-like cells by upregulating the expression of Cx37, Cx40 and Cx43 and enhancing the function of intercellular communication (Xie et al., 2013; Li et al., 2018).

### 3.6 Curcumin

Curcumin (1,7-bis(4-hydroxy-3-methoxyphenyl)-1,6-heptadiene-3,5-dione) is a phenolic natural product isolated from the roots of *Curcuma longa L*. Curcumin is reported to be a nutritional compound with a wide range of therapeutic effects and great medicinal potential. Curcumin has a variety of pharmacological effects, including anti-inflammatory, antioxidant, antiproliferative and antiangiogenic effects. It has been extensively studied in a variety of diseases, including cancer, cardiovascular disease, diabetes, arthritis, neurological diseases, and Crohn’s disease (Genchi et al., 2024; Moon, 2024).

In terms of the ability of curcumin to modify MSCs, first, curcumin can slow the ageing of MSCs. Some studies have shown that curcumin can delay ageing by activating autophagy (Deng et al., 2021), and some studies have reported that its antiaging effect is related to its concentration. Curcumin at concentrations of 1 and 5 µM is a good antioxidant that can improve the lifespan of ASCs. The mechanism involves increasing the expression of the *TERT* gene (Pirmoradi et al., 2018). Second, curcumin can promote the osteogenic differentiation of BMSCs and inhibit the formation of adipocytes, which may be related to the expression of HO-1 (Gu et al., 2012). It has also been confirmed that curcumin can inhibit the expression of Kruppel-like Factor 15 (KLF15), which may bind to the PPARγ promoter, resulting in the downregulation of PPARγ expression to inhibit the lipid-forming differentiation of hMSCs. In addition, it can also protect the mitochondrial function of BMSCs. Moreover, it can improve the tolerance of ASCs to oxidative stress damage (Wang et al., 2019). The dual effects of curcumin on stem cell survival and proliferation are related to its concentration, treatment period and type of stem cells. Therefore, these results should be applied reasonably to aid in understanding the role of curcumin in the transformation of stem cells (Attari et al., 2015).

### 3.7 Ginsenoside Rg1

Ginsenoside Rg1 belongs to the B-panaxtriol group of ginsenosides and is one of the main natural bioactive compounds of *Panax ginseng*. In traditional Chinese medicine, roots and rhizomes are the main medicinal parts of the plant. Rg1 ginsenosides are present in the stems, leaves and buds of *P. ginseng* and *Panax notoginseng* (Wu et al., 2022). In recent years, the pharmacological activity and bioavailability of ginsenoside Rg1 have been studied, and some new insights have been obtained (Gao et al., 2020; Lu et al., 2022). Ginsenoside Rg1 is a natural *ginseng* extract that has a variety of pharmacological effects, including anti-inflammatory, antioxidant and antiaging properties (Wang et al., 2021). The antiaging and antioxidant effects of Rg1 are related to the activation of the nuclear Factor E2-related Factor 2 (Nrf2) signalling pathway. Rg1 may activate the Nrf2 pathway by upregulating P62 and activating Akt to increase the interaction between P62 and KEAP1. Therefore, the activation of Akt or Nrf2 may be an important target for preventing BMSC ageing (Wang et al., 2021). In addition, studies have shown that Rg1 can reduce overactivation of the Wnt pathway in ageing cells by inhibiting the phosphorylation of GSK-3β and regulating the differentiation ability of MSCs (Wang et al., 2020). Rg1 can also promote the osteogenic differentiation of MSCs. Studies have shown that Rg1 can promote the osteogenic differentiation of BMSCs by activating the GR/BMP-2 signalling pathway (Gu et al., 2016). Other studies have shown that Rg1 can also induce the expression of cellular neurons, such as cells. The researchers cultured rat MSCs with the serum-free ginsenoside Rgl (10 μmol/L) for 3 days. Some of the cells expressed NSE, but GFAP staining was negative. The *NGF* mRNA level in the ginsenoside Rgl treatment group was significantly greater than that in the control group, suggesting that ginsenoside Rgl induced neuron-like cells to express *NGF* mRNA (Si et al., 2014).

### 3.8 Thymoquinone (TQ)

*Nigella damascena L.* is an annual herb in the *Ranunculaceae* family. TQ is the key active component of *N. damascena L.*, with a molecular formula of C_10_H_12_O_2_ and a molar mass of 164.20 g/mol (Mekhemar et al., 2020). Its anti-inflammatory, antioxidant, antibacterial and anticancer properties have broad application prospects in biomedicine. TQ-mediated stimulation preserves the pluripotency of gingival mesenchymal stem cells/progenitors (G-MSCs) and promotes the transformation of these cells into immunocompetent differentiated phenotypes by increasing TLR3 expression. This characteristic may influence the potential therapeutic application of G-MSCs (Mekhemar et al., 2022). TQ can alter angiogenesis and the mesenchymal to epithelial transformation of human breast CSCs *in vitro*. Therefore, TQ, together with antiangiogenic therapy, may be a novel therapeutic agent to inhibit breast cancer angiogenesis (Haiaty et al., 2021a; Haiaty et al., 2021b).

Acute neurodegeneration due to stroke or trauma, for example, can lead to local cell damage in the injured area, whereas chronic neurodegeneration can lead to damage to specific subtypes of neurons and widespread loss of specific groups of neurons (Qin et al., 2007). Regenerating neurons by using stem cells seems to be a useful approach, as these cells can differentiate into multiple lineages (Lunn et al., 2011). The potential of stem cells to differentiate into neuronal lineages can be enhanced by the use of various compounds. TQ is a bioactive compound with neuroprotective properties (Ishaque et al., 2022). TQ was found to induce the differentiation of MSCs and promote significant gene expression of neuronal markers, including neuronal-specific enolase (NSE), nestin, microtubule-associated protein 2 (MAP2), neurofilament light chain (Nefl), and tau, as well as astrocyte markers, Glial fibrillary acidic protein (GFAP) (Ishaque et al., 2022). Similarly, studies have shown that TQ promotes the acceleration of stem cell differentiation into osteoblasts, the mechanism of which may be related to the inhibition of NF-κB signalling (Banu, 2022), and has been shown to not alter the physical and mechanical properties of the scaffold (Rahmani-Moghadam et al., 2021).

### 3.9 Ptychotis verticillata (PV)

*Ptychotis verticillata L.* is an aromatic plant, and PV and its phytochemical components (thymol and carvacrol) have been reported as valuable medicinal candidates (El Ouariachi el et al., 2011; Seo et al., 2019). Some studies have explored the effects of PV and its compounds on the immunological characteristics of MSCs. It was first emphasized that PV extracts can maintain low immunogenicity and enhance the immunomodulatory function of MSCs. The positive effects of the essential oils thymol and carvacrol on the immune properties of MSCs will open a new field for the use of natural compounds in cell therapy (Najar et al., 2024). Like many of the compounds mentioned earlier, thymol is also concentration dependent, and in the presence of thymol (3 and 6 μg/mL), MSCs show an increased ability to inhibit T-cell responses during direct coculture. High concentrations of thymol were more effective at reducing MSC-activated T-cell responses. The incubation time also affected the inhibitory properties of MSCs pretreated with thymol, as a small reduction was observed after 5 days. Based on this evidence, we aimed to study the effects of the characteristics of the MSC immune system, PV and its compounds (Najar et al., 2024). In addition, carvacrol and thymol have been shown to alter the maturation and function of dendritic cells, as well as the T-cell response and activation.

Different plant-derived components have been shown to promote MSC migration and homing to damaged sites to enhance tissue repair and healing. The activation of the PI3K and MAPK signalling pathways and the CXCL12/CXCR4 axis and the increased expression of matrix metalloproteinases (MMPs) may stimulate extracellular matrix remodelling, thereby promoting the migration of MSCs (Maeda, 2020). Other studies have shown that thymol and carvacrol increase osteogenesis and adipogenesis in a time- and dose-dependent manner and stimulate tissue regeneration and the repair function of MSCs, and further optimization of PV compound extraction and characterization and cell processing conditions should increase their therapeutic value in combination with MSCs (Bouhtit et al., 2022).

## 4 Regulatory effects of natural bioactive compounds on MSCs

**FIGURE 3:**
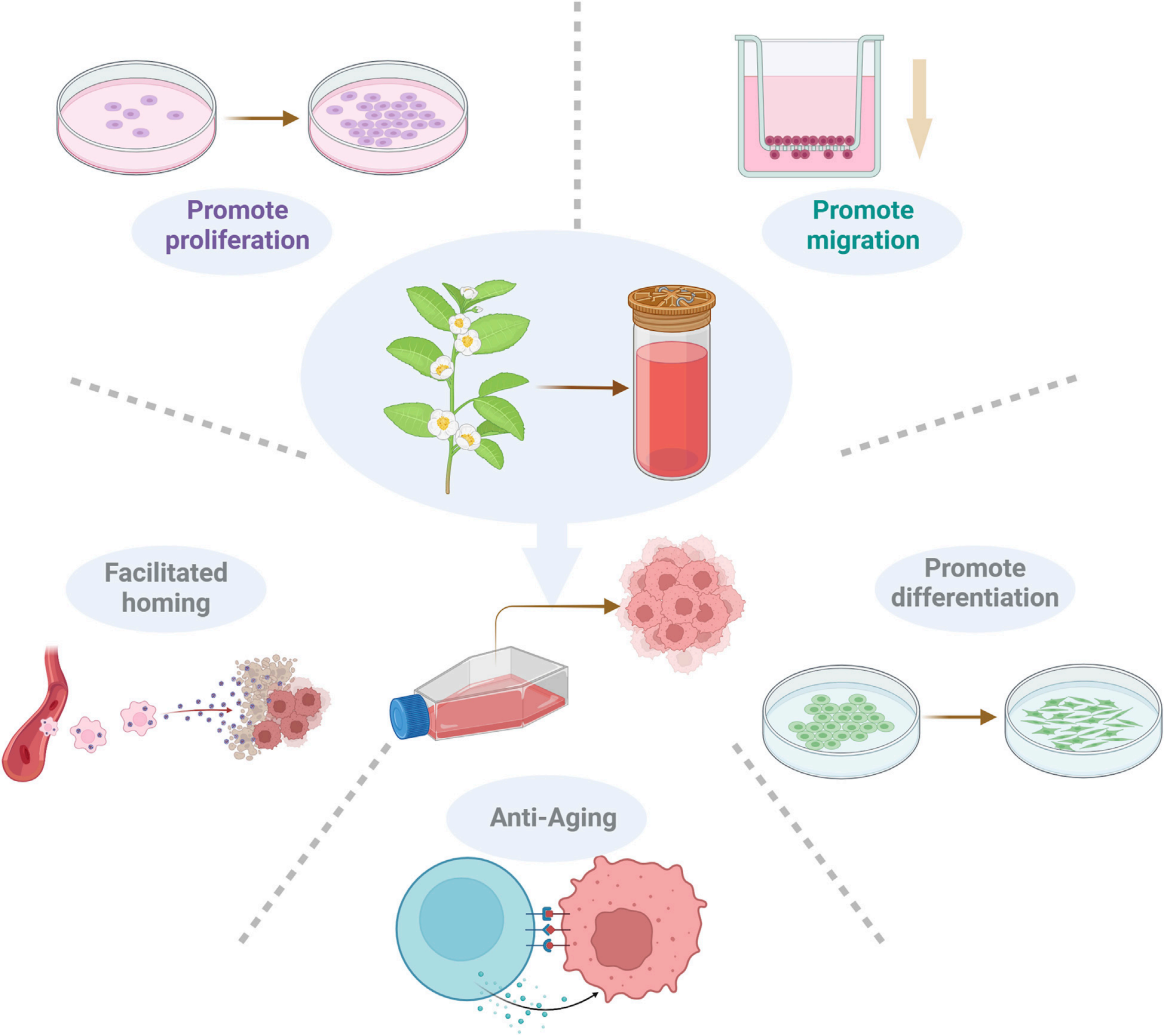
Regulatory effects of bioactive compounds on MSCs. Natural bioactive compounds can promote the diffusion, migration, homing, differentiation and antiaging effects of MSCs.

Although many natural bioactive compounds have shown great potential for improving MSCs, they involve many mechanisms and pathways, making related research complicated and confusing. In fact, we found that natural bioactive compounds promote MSC proliferation, migration and homing and promote MSC differentiation and antiaging effects (Figure 3). However, their effects can be guaranteed only under certain conditions. For example, although resveratrol can increase the self-renewal potential and pluripotency of early-passage MSCs, it also accelerates the senescence of late-passage MSCs. Therefore, cell passage and SIRT1 expression must be considered before resveratrol is used for late-passage MSCs (Yoon et al., 2015). In addition, the amount of naturally active compounds is also an important influencing factor; for example, 250 mg/kg/day is the most effective dose under the conditions where ICA has been shown to be beneficial to OP rats. However, ICA may inhibit the differentiation of MSCs into adipocytes by inhibiting the expression of *PPARγ*, *C/EBPα*, and *FABP4* mRNAs. ICA can also inhibit *Notch2* mRNA expression by inhibiting *N1ICD* expression. Therefore, further preclinical studies are needed to better define the pharmacological targets of ICA and to determine the associations between different signalling pathways (Liu et al., 2017). In the modification of MSCs by GBE, the key role of drug concentration was clearly proposed, and it was proposed that GBE improved the proliferation and osteogenesis of human BM-MSCs in a dose-dependent manner in the range of 25–75 mg/L. However, this effect was weakened or inhibited at 100 mg/L or higher (Gu et al., 2015). In summary, natural bioactive compounds have complex and context-dependent effects on MSCs.

### 4.1 Promotion of proliferation, migration and homing

Although MSCs have various advantages and good applications in regenerative medicine, they are limited in the amount of tissue they target and have challenges associated with poor migration and homing. Therefore, it is worth exploring how to promote the proliferation, migration and homing of MSCs to play a better role in clinical practice (Galipeau and Sensébé, 2018; Ullah et al., 2019). RSVL can play a positive role in the treatment of periodontitis by promoting the proliferation of MSCs (Jiang et al., 2023). Furthermore, AS-IV improved ADSC migration (Wang et al., 2022). *In vivo* animal experiments have confirmed that Rg1 promotes the homing of rabbit BMSCs to myocardial tissue (Wang et al., 2005). These changes reduced the myocardial infarct size and improved cardiac function. Some of them are obviously related to their concentration, so special attention should be given to this point when they are used (Gu et al., 2015).

### 4.2 Promotion of differentiation

In some pathological conditions, such as ageing, osteoporosis and some bone defects, the osteogenic ability of BMSCs is significantly inhibited. Therefore, methods that can modulate the osteogenic differentiation of BMSCs to manage and treat these diseases are needed (Houdek et al., 2016; Zhang et al., 2021). In addition to RSVL, GBE, Tan IIA, and AGS-IV can promote the osteogenic differentiation of MSCs, as mentioned above, and studies have shown that walnut leaf extract regulates the osteogenic differentiation and autophagy of hBMSCs through the BMP2/Smad/Runx2 and Wnt/β-catenin pathways (Pang et al., 2022). Unlike the concentration dependence mentioned earlier, this study confirmed that different concentrations of walnut leaf extract did not have a significant effect on cell proliferation, indicating a reliable safety profile of hBMSCs treated with walnut leaf extract (Pang et al., 2022). Quercetin can activate the Wnt/β-catenin pathway through the H19/miR-625-5p axis and promote the osteogenic differentiation of BMSCs (Bian et al., 2021). In summary, various natural bioactive compounds have been demonstrated to enhance the differentiation of MSCs through distinct signalling pathways.

### 4.3 Anti-aging

Cellular senescence is the result of cumulative changes in cellular structure and function. Its function manifests as reduced oxidative phosphorylation, a slowed respiratory rate, and reduced enzyme activity and receptor protein levels, resulting in decreased cell function and inhibited cell proliferation (Young et al., 2019). Studies have shown that senescent cells and the *VDR* gene expression interference system can reduce cell viability, proliferation and bone differentiation ability. MSCs inevitably encounter the problem of ageing, but studies have shown that after the addition of Astragalus, the viability and osteogenic ability of BMSCs are significantly increased. Among them, the expression levels of FGF23, Klotho and CYP24A1 decreased, whereas the expression level of CYP27B1 increased, and the effect became more obvious with increasing Astragalus concentration. It has been confirmed that astragalus can inhibit the ageing of BMSCs and improve their osteogenic ability by regulating the VD-FGF23-Klotho pathway (Pu et al., 2020). As mentioned earlier, curcumin can alleviate the ageing of BMSCs by activating autophagy, and curcumin is also a good antioxidant that can increase the lifespan of ASCs (Liu et al., 2015; Ayadilord et al., 2022). In summary, regulating ageing-related pathways and promoting autophagy are key to improving the effects of natural bioactive compounds on MSCs. The effects of natural bioactive compounds themselves, such as antioxidation, are the potential basis for these effects.

## 5 Discussion

With the continuous development of cell therapy, researchers are striving to achieve the goal of effective repair and regeneration after tissue damage. The application of allogeneic human stem cells benefits from the immune tolerance of specific types of stem cells. In this context, the use of MSCs as promising tools for cellular immunotherapy is being explored for the treatment of a variety of diseases (Hussen et al., 2024). However, the clinical application of MSCs also faces many challenges. In the process of preparing MSC products, the main challenges include the following: 1) the heterogeneity of MSCs is caused by donor differences such as health status, genetics, gender and age. 2) The dry stability and differentiation ability of MSCs isolated from different sources (such as bone marrow, adipose tissue, the umbilical cord, or muscle) differ.3) The amplification ability was different under different culture conditions. In stem cell applications, the application of MSCs may be limited for the following reasons: 1) Challenges remain regarding the influence of the homing or migration ability of MSCs under different administration routes (local/systemic), injection sites, infusion times, and cell carrier materials. 2) Immunocompatibility between donors and recipients is key to reducing the risk of rejection. 3) The complex active components released by MSCs depend on the host microenvironment (inflammatory state, hypoxia, and ECM), which can lead to highly variable factors shaping the different functions of MSCs (Zhou et al., 2021). Therefore, improving the treatment efficiency of MSCs on the premise of ensuring safety, which has been discussed in the relevant literature (Seo et al., 2019), is imperative.

Natural bioactive compounds such as RSVL, GBE and curcumin can regulate the survival and proliferation of MSCs, but their effects on proliferation are closely related to their concentration, so caution should be taken. ICA and RSVL can promote osteogenic differentiation and inhibit lipid differentiation via different mechanisms, and research on nonbone tissue differentiation is insufficient. Tan IIA and AGS-IV can promote MSC migration and homing, but few relevant studies exist. Curcumin and Rg1 can inhibit MSC ageing, but such studies are scarce. These compounds have multitarget and multilevel effects, have different effects on the improvement of MSCs, and may receive more attention in the future.

Although the effects of many bioactive compounds on MSCs have been discussed above, several issues warrant attention. For example, not all concentrations of bioactive compounds can positively modify MSCs, and they are not all concentration dependent; each has its own characteristics. In addition, relatively few *in vivo* experiments have investigated the effects of natural bioactive compounds on MSCs, which may be the direction of our attention in the future. Moreover, another challenge is that most of the natural bioactive compounds mentioned in the literature are only 98% or 99% pure. However, their application in the field of stem cell research is promising, and we hope to further identify their purity, remove impurities, and achieve better promotion and application of these compounds in the future (Li et al., 2024).

## 6 Conclusion

Different types of natural bioactive compounds have different effects on the improvement of MSCs, such as promoting their proliferation and differentiation, migration and homing, and antiaging effects. It is expected that improved MSCs can provide new ideas for disease treatment and regenerative medicine in the future.

## References

[B1] Abbott A. (2025). Stem cells head to the clinic: treatments for cancer, diabetes and Parkinson's disease could soon be here. Nature 637 (8044), 18–20. 10.1038/d41586-024-04160-0 39707007

[B2] Al-Azab M. Idiiatullina E. Safi M. Hezam K. (2023). Enhancers of mesenchymal stem cell stemness and therapeutic potency. Biomed. Pharmacother. 162, 114356. 10.1016/j.biopha.2023.114356 37040673

[B3] Al-Azab M. Safi M. Idiiatullina E. Al-Shaebi F. Zaky M. Y. (2022). Aging of mesenchymal stem cell: machinery, markers, and strategies of fighting. Cell Mol. Biol. Lett. 27 (1), 69. 10.1186/s11658-022-00366-0 35986247 PMC9388978

[B4] Al-Ghadban S. Artiles M. Bunnell B. A. (2022). Adipose stem cells in regenerative medicine: looking forward. Front. Bioeng. Biotechnol. 9, 837464. 10.3389/fbioe.2021.837464 35096804 PMC8792599

[B5] Al-Sammarraie S. H. A. Ayaz-Güner Ş. Acar M. B. Şimşek A. Sınıksaran B. S. Bozalan H. D. (2024). Mesenchymal stem cells from adipose tissue prone to lose their stemness associated markers in obesity related stress conditions. Sci. Rep. 14 (1), 19702. 10.1038/s41598-024-70127-w 39181924 PMC11344827

[B6] Ansari M. A. Khan F. B. Safdari H. A. Almatroudi A. Alzohairy M. A. Safdari M. (2021). Prospective therapeutic potential of Tanshinone IIA: an updated overview. Pharmacol. Res. 164, 105364. 10.1016/j.phrs.2020.105364 33285229

[B7] Attari F. Zahmatkesh M. Aligholi H. Mehr S. E. Sharifzadeh M. Gorji A. (2015). Curcumin as a double-edged sword for stem cells: dose, time and cell type-specific responses to curcumin. Daru 23 (1), 33. 10.1186/s40199-015-0115-8 26063234 PMC4466857

[B8] Ayadilord M. Saharkhiz M. Naseri M. Emadian Razavi F. (2022). Expression of immunomodulatory and tissue regenerative biomarkers in human dental pulp derived-mesenchymal stem cells treated with curcumin: an *in vitro* study. Mol. Biol. Rep. 49 (6), 4411–4420. 10.1007/s11033-022-07278-4 35301656

[B9] Banu I. (2022). The active compound thymoquinone alters chondrogenic differentiation of human mesenchymal stem cells via modulation of intracellular signaling. Medeni. Med. J. 37 (1), 1–12. 10.4274/MMJ.galenos.2022.68915 35306780 PMC8939446

[B10] Barone L. Palano M. T. Gallazzi M. Cucchiara M. Rossi F. Borgese M. (2023). Adipose mesenchymal stem cell-derived soluble factors, produced under hypoxic condition, efficiently support *in vivo* angiogenesis. Cell Death Discov. 9 (1), 174. 10.1038/s41420-023-01464-4 37221171 PMC10205717

[B11] Bi Z. Zhang W. Yan X. (2022). Anti-inflammatory and immunoregulatory effects of icariin and icaritin. Biomed. Pharmacother. 151, 113180. 10.1016/j.biopha.2022.113180 35676785

[B12] Bian W. Xiao S. Yang L. Chen J. Deng S. (2021). Quercetin promotes bone marrow mesenchymal stem cell proliferation and osteogenic differentiation through the H19/miR-625-5p axis to activate the Wnt/β-catenin pathway. BMC Complement. Med. Ther. 21 (1), 243. 10.1186/s12906-021-03418-8 34592982 PMC8485455

[B13] Bouhtit F. Najar M. Rahmani S. Melki R. Najimi M. Sadki K. (2022). Bioscreening and pre-clinical evaluation of the impact of bioactive molecules from Ptychotis verticillata on the multilineage potential of mesenchymal stromal cells towards immune- and inflammation-mediated diseases. Inflamm. Res. 71 (7-8), 887–898. 10.1007/s00011-022-01573-3 35716172

[B14] Caldarelli I. Speranza M. C. Bencivenga D. Tramontano A. Borgia A. Pirozzi A. V. (2015). Resveratrol mimics insulin activity in the adipogenic commitment of human bone marrow mesenchymal stromal cells. Int. J. Biochem. Cell Biol. 60, 60–72. 10.1016/j.biocel.2014.12.011 25562512

[B15] Cao Y. Boss A. L. Bolam S. M. Munro J. T. Crawford H. Dalbeth N. (2024). *In vitro* cell surface marker expression on mesenchymal stem cell cultures does not reflect their *ex vivo* phenotype. Stem Cell Rev. Rep. 20 (6), 1656–1666. 10.1007/s12015-024-10743-1 38837115 PMC11319515

[B16] Cao Y. Lv Q. Li Y. (2021). Astragaloside IV improves tibial defect in rats and promotes proliferation and osteogenic differentiation of hBMSCs through MiR-124-3p.1/STAT3 Axis. J. Nat. Prod. 84 (2), 287–297. 10.1021/acs.jnatprod.0c00975 33464097

[B17] Cawthorn W. P. Scheller E. L. MacDougald O. A. (2012). Adipose tissue stem cells: the great WAT hope. Trends Endocrinol. Metab. 23 (6), 270–277. 10.1016/j.tem.2012.01.003 22417866 PMC3367055

[B171] Chen S. Liang H. Ji Y. Kou H. Zhang C. Shang G. (2021). Curcumin Modulates the Crosstalk Between Macrophages and Bone Mesenchymal Stem Cells to Ameliorate Osteogenesis[J]. Front. Cell. Dev. Biol. 9, 634650. 10.3389/fcell.2021.634650 33634135 PMC7900185

[B18] Chen W. Wang C. Yang Z. X. Zhang F. Wen W. Schaniel C. (2023). Reprogramming of human peripheral blood mononuclear cells into induced mesenchymal stromal cells using non-integrating vectors. Commun. Biol. 6 (1), 393. 10.1038/s42003-023-04737-x 37041280 PMC10090171

[B19] Dai C. Liu Y. Dong Z. (2017). Tanshinone I alleviates motor and cognitive impairments via suppressing oxidative stress in the neonatal rats after hypoxic-ischemic brain damage. Mol. Brain 10 (1), 52. 10.1186/s13041-017-0332-9 29137683 PMC5686905

[B20] Dai Z. Li Y. Quarles L. D. Song T. Pan W. Zhou H. (2007). Resveratrol enhances proliferation and osteoblastic differentiation in human mesenchymal stem cells via ER-dependent ERK1/2 activation. Phytomedicine 14 (12), 806–814. 10.1016/j.phymed.2007.04.003 17689939

[B21] Daneshmandi L. Shah S. Jafari T. Bhattacharjee M. Momah D. Saveh-Shemshaki N. (2020). Emergence of the stem cell secretome in regenerative engineering. Trends Biotechnol. 38 (12), 1373–1384. 10.1016/j.tibtech.2020.04.013 32622558 PMC7666064

[B22] Deng J. Ouyang P. Li W. Zhong L. Gu C. Shen L. (2021). Curcumin alleviates the senescence of canine bone marrow mesenchymal stem cells during *in vitro* expansion by activating the autophagy pathway. Int. J. Mol. Sci. 22 (21), 11356. 10.3390/ijms222111356 34768788 PMC8583405

[B23] Ding M. Shen Y. Wang P. Xie Z. Xu S. Zhu Z. (2018). Exosomes isolated from human umbilical cord mesenchymal stem cells alleviate neuroinflammation and reduce amyloid-beta deposition by modulating microglial activation in Alzheimer's disease. Neurochem. Res. 43 (11), 2165–2177. 10.1007/s11064-018-2641-5 30259257

[B24] Doktor F. Antounians L. Figueira R. L. Khalaj K. Duci M. Zani A. (2025). Amniotic fluid stem cell extracellular vesicles as a novel fetal therapy for pulmonary hypoplasia: a review on mechanisms and translational potential. Stem Cells Transl. Med. 14 (1), szae095. 10.1093/stcltm/szae095 39823257 PMC11740888

[B25] Eirin A. Thaler R. Glasstetter L. M. Xing L. Zhu X. Y. Osborne A. C. (2024). Obesity-driven mitochondrial dysfunction in human adipose tissue-derived mesenchymal stem/stromal cells involves epigenetic changes. Cell Death Dis. 15 (6), 387. 10.1038/s41419-024-06774-8 38824145 PMC11144257

[B26] Eiro N. Fraile M. Escudero-Cernuda S. Sendon-Lago J. Gonzalez L. O. Fernandez-Sánchez M. L. (2024). Synergistic effect of human uterine cervical mesenchymal stem cell secretome and paclitaxel on triple negative breast cancer. Stem Cell Res. Ther. 15 (1), 121. 10.1186/s13287-024-03717-0 38664697 PMC11044487

[B27] El Ouariachi el M. Tomi P. Bouyanzer A. Hammouti B. Desjobert J. M. Costa J. (2011). Chemical composition and antioxidant activity of essential oils and solvent extracts of Ptychotis verticillata from Morocco. Food Chem. Toxicol. 49 (2), 533–536. 10.1016/j.fct.2010.11.019 21093522

[B28] Feng J. Liao L. Xu F. Zhang L. Zhang J. (2022). Combination of stem cells with Chinese herbs for secondary depression in neurodegenerative diseases based on traditional Chinese medicine theories. Evid. Based Complement. Altern. Med. 2022, 1–17. 10.1155/2022/6847917 PMC8913071 35280507

[B29] Fu X. Liu G. Halim A. Ju Y. Luo Q. Song A. G. (2019). Mesenchymal stem cell migration and tissue repair. Cells 8 (8), 784. 10.3390/cells8080784 31357692 PMC6721499

[B30] Galipeau J. Sensébé L. (2018). Mesenchymal stromal cells: clinical challenges and therapeutic opportunities. Cell Stem Cell 22 (6), 824–833. 10.1016/j.stem.2018.05.004 29859173 PMC6434696

[B31] Gao Y. Li J. Wang J. Li X. Li J. Chu S. (2020). Ginsenoside Rg1 prevent and treat inflammatory diseases: a review. Int. Immunopharmacol. 87, 106805. 10.1016/j.intimp.2020.106805 32731179

[B32] Ge Y. Wu J. Zhang L. Huang N. Luo Y. (2024). A new strategy for the regulation of neuroinflammation: exosomes derived from mesenchymal stem cells. Cell Mol. Neurobiol. 44 (1), 24. 10.1007/s10571-024-01460-x 38372822 PMC10876823

[B33] Genchi G. Lauria G. Catalano A. Carocci A. Sinicropi M. S. (2024). Neuroprotective effects of curcumin in neurodegenerative diseases. Neurodegener. Dis. 13 (11), 1774. 10.3390/foods13111774 PMC11172163 38891002

[B34] Geng Y. W. Zhang Z. Liu M. Y. Hu W. P. (2017). Differentiation of human dental pulp stem cells into neuronal by resveratrol. Cell Biol. Int. 41 (12), 1391–1398. 10.1002/cbin.10835 28782906

[B35] Gopalarethinam J. Nair A. P. Iyer M. Vellingiri B. Subramaniam M. D. (2023). Advantages of mesenchymal stem cell over the other stem cells. Acta histochem. 125 (4), 152041. 10.1016/j.acthis.2023.152041 37167794

[B36] Gou Y. Huang Y. Luo W. Li Y. Zhao P. Zhong J. (2024). Adipose-derived mesenchymal stem cells (MSCs) are a superior cell source for bone tissue engineering. Bioact. Mater 34, 51–63. 10.1016/j.bioactmat.2023.12.003 38186960 PMC10770370

[B37] Gu Q. Cai Y. Huang C. Shi Q. Yang H. (2012). Curcumin increases rat mesenchymal stem cell osteoblast differentiation but inhibits adipocyte differentiation. Pharmacogn. Mag. 8 (31), 202–208. 10.4103/0973-1296.99285 23060694 PMC3466455

[B38] Gu Q. Chen C. Zhang Z. Wu Z. Fan X. Zhang Z. (2015). Ginkgo biloba extract promotes osteogenic differentiation of human bone marrow mesenchymal stem cells in a pathway involving Wnt/β-catenin signaling. Pharmacol. Res. 97, 70–78. 10.1016/j.phrs.2015.04.004 25917209

[B39] Gu Y. Zhou J. Wang Q. Fan W. Yin G. (2016). Ginsenoside Rg1 promotes osteogenic differentiation of rBMSCs and healing of rat tibial fractures through regulation of GR-dependent BMP-2/SMAD signaling. Sci. Rep. 6, 25282. 10.1038/srep25282 27141994 PMC4855182

[B40] Gugliandolo A. Bramanti P. Mazzon E. (2020). Activation of Nrf2 by natural bioactive compounds: a promising approach for stroke? Int. J. Mol. Sci. 21 (14), 4875. 10.3390/ijms21144875 32664226 PMC7402299

[B41] Haiaty S. Rashidi M. R. Akbarzadeh M. Bazmani A. Mostafazadeh M. Nikanfar S. (2021a). Correction to: thymoquinone inhibited vasculogenic capacity and promoted mesenchymal-epithelial transition of human breast cancer stem cells. BMC Complement. Med. Ther. 21 (1), 266. 10.1186/s12906-021-03414-y 34696750 PMC8543957

[B42] Haiaty S. Rashidi M. R. Akbarzadeh M. Bazmani A. Mostafazadeh M. Nikanfar S. (2021b). Thymoquinone inhibited vasculogenic capacity and promoted mesenchymal-epithelial transition of human breast cancer stem cells. BMC Complement. Med. Ther. 21 (1), 83. 10.1186/s12906-021-03246-w 33663486 PMC7931333

[B43] Han H. Chen B. T. Liu Y. Wang Y. Xing L. Wang H. (2024). Engineered stem cell-based strategy: a new paradigm of next-generation stem cell product in regenerative medicine. J. Control Release 365, 981–1003. 10.1016/j.jconrel.2023.12.024 38123072

[B44] Han Y. Yang J. Fang J. Zhou Y. Candi E. Wang J. (2022). The secretion profile of mesenchymal stem cells and potential applications in treating human diseases. Signal Transduct. Target Ther. 7 (1), 92. 10.1038/s41392-022-00932-0 35314676 PMC8935608

[B45] Hazrati A. Malekpour K. Khorramdelazad H. Rajaei S. Hashemi S. M. (2024). Therapeutic and immunomodulatory potentials of mesenchymal stromal/stem cells and immune checkpoints related molecules. Biomark. Res. 12 (1), 35. 10.1186/s40364-024-00580-2 38515166 PMC10958918

[B46] He C. Wang Z. Shi J. (2020). Pharmacological effects of icariin. Adv. Pharmacol. 87, 179–203. 10.1016/bs.apha.2019.10.004 32089233

[B47] Houdek M. T. Wyles C. C. Packard B. D. Terzic A. Behfar A. Sierra R. J. (2016). Decreased osteogenic activity of mesenchymal stem cells in patients with corticosteroid-induced osteonecrosis of the femoral head. J. Arthroplasty 31 (4), 893–898. 10.1016/j.arth.2015.08.017 26404846

[B48] Hsieh T. P. Sheu S. Y. Sun J. S. Chen M. H. (2011). Icariin inhibits osteoclast differentiation and bone resorption by suppression of MAPKs/NF-κB regulated HIF-1α and PGE(2) synthesis. Phytomedicine 18 (2-3), 176–185. 10.1016/j.phymed.2010.04.003 20554188

[B49] Hu C. Li L. (2019). The application of resveratrol to mesenchymal stromal cell-based regenerative medicine. Stem Cell Res. Ther. 10 (1), 307. 10.1186/s13287-019-1412-9 31623691 PMC6798337

[B50] Hu D. Wang H. J. Yu L. H. Guan Z. R. Jiang Y. P. Hu J. H. (2023). The role of Ginkgo Folium on antitumor: bioactive constituents and the potential mechanism. J. Ethnopharmacol. 321, 117202. 10.1016/j.jep.2023.117202 37742878

[B51] Huang J. Huang N. Mao Q. Shi J. Qiu Y. (2023). Natural bioactive compounds in Alzheimer's disease: from the perspective of type 3 diabetes mellitus. Front. Aging Neurosci. 15, 1130253. 10.3389/fnagi.2023.1130253 37009462 PMC10062602

[B52] Huang J. Huang N. Xu S. Luo Y. Li Y. Jin H. (2020). Signaling mechanisms underlying inhibition of neuroinflammation by resveratrol in neurodegenerative diseases. J. Nutr. Biochem. 88, 108552. 10.1016/j.jnutbio.2020.108552 33220405

[B53] Huang J. M. Bao Y. Xiang W. Jing X. Z. Guo J. C. Yao X. D. (2017). Icariin regulates the bidirectional differentiation of bone marrow mesenchymal stem cells through canonical Wnt signaling pathway. Evid. Based Complement. Altern. Med. 2017, 8085325. 10.1155/2017/8085325 PMC5763109 29445413

[B54] Huang N. Huang J. Feng F. Ba Z. Li Y. Luo Y. (2022). Tanshinone ΙΙA-incubated mesenchymal stem cells inhibit lipopolysaccharide-induced inflammation of N9 cells through TREM2 signaling pathway. Stem Cells Int. 2022, 1–7. 10.1155/2022/9977610 PMC8916899 35283996

[B55] Huang N. Huang W. Wu J. Long S. Luo Y. Huang J. (2024). Possible opportunities and challenges for traditional Chinese medicine research in 2035. Front. Pharmacol. 15, 1426300. 10.3389/fphar.2024.1426300 38974044 PMC11224461

[B56] Huang N. Li Y. Zhou Y. Zhou Y. Feng F. Shi S. (2019). Neuroprotective effect of tanshinone IIA-incubated mesenchymal stem cells on Aβ(25-35)-induced neuroinflammation. Behav. Brain Res. 365, 48–55. 10.1016/j.bbr.2019.03.001 30831140

[B57] Huang Y. Wang J. Cai J. Qiu Y. Zheng H. Lai X. (2018). Targeted homing of CCR2-overexpressing mesenchymal stromal cells to ischemic brain enhances post-stroke recovery partially through PRDX4-mediated blood-brain barrier preservation. Theranostics 8 (21), 5929–5944. 10.7150/thno.28029 30613272 PMC6299433

[B58] Hussen B. M. Taheri M. Yashooa R. K. Abdullah G. H. Abdullah S. R. Kheder R. K. (2024). Revolutionizing medicine: recent developments and future prospects in stem-cell therapy. 110(12), 8002–8024. 10.1097/js9.0000000000002109 PMC11634165 39497543

[B59] Ishaque A. Khan I. Salim A. Qazi R. E. Malick T. S. Adli D. S. H. (2022). Effect of α-pinene and thymoquinone on the differentiation of bone marrow mesenchymal stem cells into neuroprogenitor cells. Bioimpacts 12 (2), 147–154. 10.34172/bi.2021.23634 35411294 PMC8905589

[B60] Jahan S. Kumar D. Singh S. Kumar V. Srivastava A. Pandey A. (2018a). Resveratrol prevents the cellular damages induced by monocrotophos via PI3K signaling pathway in human cord blood mesenchymal stem cells. Mol. Neurobiol. 55 (11), 8278–8292. 10.1007/s12035-018-0986-z 29526017

[B61] Jahan S. Singh S. Srivastava A. Kumar V. Kumar D. Pandey A. (2018b). PKA-GSK3β and β-catenin signaling play a critical role in trans-resveratrol mediated neuronal differentiation in human cord blood stem cells. Mol. Neurobiol. 55 (4), 2828–2839. 10.1007/s12035-017-0539-x 28455695

[B62] Jiang H. Ni J. Hu L. Xiang Z. Zeng J. Shi J. (2023). Resveratrol may reduce the degree of periodontitis by regulating ERK pathway in gingival-derived MSCs. Int. J. Mol. Sci. 24 (14), 11294. 10.3390/ijms241411294 37511053 PMC10378998

[B63] Jiao F. Tang W. Huang H. Zhang Z. Liu D. Zhang H. (2018). Icariin promotes the migration of BMSCs *in vitro* and *in vivo* via the MAPK signaling pathway. Stem Cells Int. 2018, 1–9. 10.1155/2018/2562105 PMC6167584 30319696

[B64] Joe I. S. Jeong S. G. Cho G. W. (2015). Resveratrol-induced SIRT1 activation promotes neuronal differentiation of human bone marrow mesenchymal stem cells. Neurosci. Lett. 584, 97–102. 10.1016/j.neulet.2014.10.024 25459285

[B65] Kaiser E. E. Waters E. S. Yang X. Fagan M. M. Scheulin K. M. Sneed S. E. (2022). Tanshinone IIA-loaded nanoparticle and neural stem cell therapy enhances recovery in a pig ischemic stroke model. Stem Cells Transl. Med. 11 (10), 1061–1071. 10.1093/stcltm/szac062 36124817 PMC9585947

[B66] Khattar S. Khan S. A. Zaidi S. A. A. Darvishikolour M. Farooq U. Naseef P. P. (2022). Resveratrol from dietary supplement to a drug candidate: an assessment of potential. Pharm. (Basel). 15 (8), 957. 10.3390/ph15080957 PMC9412308 36015105

[B67] Kim J. H. Kim D. H. Jo S. Cho M. J. Cho Y. R. Lee Y. J. (2022). Immunomodulatory functional foods and their molecular mechanisms. Exp. Mol. Med. 54 (1), 1–11. 10.1038/s12276-022-00724-0 35079119 PMC8787967

[B68] Lakshminarayanan A. Kannan S. Kuppusamy M. K. Sankaranarayanan K. Godla U. Punnoose A. M. (2025). The effect of curcumin, catechin and resveratrol on viability, proliferation and cytotoxicity of human umbilical cord wharton’s jelly derived mesenchymal stem cells. Tissue Cell 93, 102742. 10.1016/j.tice.2025.102742 39874919

[B69] Lan T. Luo M. Wei X. (2021). Mesenchymal stem/stromal cells in cancer therapy. J. Hematol. Oncol. 14 (1), 195. 10.1186/s13045-021-01208-w 34789315 PMC8596342

[B70] Li L. Li X. Han R. Wu M. Ma Y. Chen Y. (2023). Therapeutic potential of Chinese medicine for endogenous neurogenesis: a promising candidate for stroke treatment. Pharm. (Basel) 16 (5), 706. 10.3390/ph16050706 PMC10221149 37242489

[B71] Li M. Jiang Y. Hou Q. Zhao Y. Zhong L. Fu X. (2022). Potential pre-activation strategies for improving therapeutic efficacy of mesenchymal stem cells: current status and future prospects. Stem Cell Res. Ther. 13 (1), 146. 10.1186/s13287-022-02822-2 35379361 PMC8981790

[B72] Li N. Gao J. Mi L. Zhang G. Zhang L. Zhang N. (2020). Synovial membrane mesenchymal stem cells: past life, current situation, and application in bone and joint diseases. Stem Cell Res. Ther. 11 (1), 381. 10.1186/s13287-020-01885-3 32894205 PMC7487958

[B73] Li W. Xiang Z. Yu W. Huang X. Jiang Q. Abumansour A. (2024). Natural compounds and mesenchymal stem cells: implications for inflammatory-impaired tissue regeneration. Stem Cell Res. Ther. 15 (1), 34. 10.1186/s13287-024-03641-3 38321524 PMC10848428

[B74] Li Z. Zhang S. Cao L. Li W. Ye Y. C. Shi Z. X. (2018). Tanshinone IIA and Astragaloside IV promote the angiogenesis of mesenchymal stem cell-derived endothelial cell-like cells via upregulation of Cx37, Cx40 and Cx43. Exp. Ther. Med. 15 (2), 1847–1854. 10.3892/etm.2017.5636 29434774 PMC5776521

[B75] Liang Y. Chen B. Liang D. Quan X. Gu R. Meng Z. (2023). Pharmacological effects of astragaloside IV: a review. Molecules 28 (16), 6118. 10.3390/molecules28166118 37630371 PMC10458270

[B76] Lim R. Z. L. Li L. Yong E. L. Chew N. (2018). STAT-3 regulation of CXCR4 is necessary for the prenylflavonoid Icaritin to enhance mesenchymal stem cell proliferation, migration and osteogenic differentiation. Biochim. Biophys. Acta Gen. Subj. 1862 (7), 1680–1692. 10.1016/j.bbagen.2018.04.016 29679717

[B77] Lin Z. Wu Y. Xu Y. Li G. Li Z. Liu T. (2022). Mesenchymal stem cell-derived exosomes in cancer therapy resistance: recent advances and therapeutic potential. Mol. Cancer 21 (1), 179. 10.1186/s12943-022-01650-5 36100944 PMC9468526

[B78] Liu H. Xiong Y. Zhu X. Gao H. Yin S. Wang J. (2017). Icariin improves osteoporosis, inhibits the expression of PPARγ, C/EBPα, FABP4 mRNA, N1ICD and jagged1 proteins, and increases Notch2 mRNA in ovariectomized rats. Exp. Ther. Med. 13 (4), 1360–1368. 10.3892/etm.2017.4128 28413478 PMC5377361

[B79] Liu J. Zhu P. Song P. Xiong W. Chen H. Peng W. (2015). Pretreatment of adipose derived stem cells with curcumin facilitates myocardial recovery via antiapoptosis and angiogenesis. Stem Cells Int. 2015, 1–12. 10.1155/2015/638153 PMC4436501 26074974

[B80] Liu X. Niu Y. Xie W. Wei D. Du Q. (2019). Tanshinone IIA promotes osteogenic differentiation of human periodontal ligament stem cells via ERK1/2-dependent Runx2 induction. Am. J. Transl. Res. 11 (1), 340–350. 30787991 PMC6357334

[B81] Liu Y. L. Zhou Y. Sun L. Wen J. T. Teng S. J. Yang L. (2014). Protective effects of Gingko biloba extract 761 on myocardial infarction via improving the viability of implanted mesenchymal stem cells in the rat heart. Mol. Med. Rep. 9 (4), 1112–1120. 10.3892/mmr.2014.1959 24549494

[B82] Lu J. Cheng H. Chen K. Zhang F. (2025). From bench to bedside: future prospects in stem cell therapy for diabetes. J. Transl. Med. 23 (1), 72. 10.1186/s12967-024-06019-4 39815257 PMC11734228

[B83] Lu J. Liu Z. Shu M. Zhang L. Xia W. Tang L. (2021). Human placental mesenchymal stem cells ameliorate chemotherapy-induced damage in the testis by reducing apoptosis/oxidative stress and promoting autophagy. Stem Cell Res. Ther. 12 (1), 199. 10.1186/s13287-021-02275-z 33743823 PMC7981860

[B84] Lu J. Wang X. Wu A. Cao Y. Dai X. Liang Y. (2022). Ginsenosides in central nervous system diseases: pharmacological actions, mechanisms, and therapeutics. Phytother. Res. 36 (4), 1523–1544. 10.1002/ptr.7395 35084783

[B85] Lunn J. S. Sakowski S. A. Hur J. Feldman E. L. (2011). Stem cell technology for neurodegenerative diseases. Ann. Neurol. 70 (3), 353–361. 10.1002/ana.22487 21905078 PMC3177143

[B86] Lv Y. J. Yang Y. Sui B. D. Hu C. H. Zhao P. Liao L. (2018). Resveratrol counteracts bone loss via mitofilin-mediated osteogenic improvement of mesenchymal stem cells in senescence-accelerated mice. Theranostics 8 (9), 2387–2406. 10.7150/thno.23620 29721087 PMC5928897

[B87] Maeda A. (2020). Recruitment of mesenchymal stem cells to damaged sites by plant-derived components. Front. Cell Dev. Biol. 8, 437. 10.3389/fcell.2020.00437 32582713 PMC7295908

[B88] Mahmoudi F. Jalayeri M. H. T. Montaseri A. MohamedKhosroshahi L. Baradaran B. (2024). Microbial natural compounds and secondary metabolites as Immunomodulators: a review. Int. J. Biol. Macromol. 278 (Pt 3), 134778. 10.1016/j.ijbiomac.2024.134778 39153680

[B89] Masuda K. Han X. Kato H. Sato H. Zhang Y. Sun X. (2021). Dental pulp-derived mesenchymal stem cells for modeling genetic disorders. Int. J. Mol. Sci. 22 (5), 2269. 10.3390/ijms22052269 33668763 PMC7956585

[B90] Matsuzaka Y. Yashiro R. (2024). Current strategies and therapeutic applications of mesenchymal stem cell-based drug delivery. Pharm. (Basel) 17 (6), 707. 10.3390/ph17060707 PMC11206583 38931374

[B91] Mekhemar M. Hassan Y. Dörfer C. (2020). Nigella sativa and thymoquinone: a natural blessing for periodontal therapy. Antioxidants (Basel) 9 (12), 1260. 10.3390/antiox9121260 33322636 PMC7764221

[B92] Mekhemar M. Tölle J. Hassan Y. Dörfer C. El-Sayed K. F. (2022). Thymoquinone-mediated modulation of toll-like receptors and pluripotency factors in gingival mesenchymal stem/progenitor cells. Cells 11 (9), 1452. 10.3390/cells11091452 35563755 PMC9101758

[B93] Moon D.-O. (2024). Curcumin in cancer and inflammation: an in-depth exploration of molecular interactions, therapeutic potentials, and the role in disease management. Management 25 (5), 2911. 10.3390/ijms25052911 PMC10932100 38474160

[B94] Mousavi S. N. Hosseinikia M. Yousefi Rad E. Saboori S. J. P. R. (2022). Beneficial effects of Ginkgo biloba leaf extract on inflammatory markers: a systematic review and meta‐analysis of the clinical trials. 36(9), 3459–3469. 10.1002/ptr.7544 35781715

[B95] Najar M. Bouhtit F. Rahmani S. Bouali A. Melki R. Najimi M. (2024). The immunogenic profile and immunomodulatory function of mesenchymal stromal/stem cells in the presence of Ptychotis verticillata. Heliyon 10 (3), e24822. 10.1016/j.heliyon.2024.e24822 38317994 PMC10838760

[B96] Ng T. W. Tan P. P. S. Lim H. M. Kumar D. N. Zain S. M. Low T. Y. (2022). Potential medicinal herb for cardiovascular health: a comprehensive review on salviae miltiorrhizae. 51(1), 1–20. 10.55230/mabjournal.v51i1.2056

[B97] Nie L. Yao D. Chen S. Wang J. Pan C. Wu D. (2023). Directional induction of neural stem cells, a new therapy for neurodegenerative diseases and ischemic stroke. Cell Death Discov. 9 (1), 215. 10.1038/s41420-023-01532-9 37393356 PMC10314944

[B98] Niu Y. Chen Y. Xu H. Wang Q. Xue C. Zhu R. (2020). Astragaloside IV promotes antiphotoaging by enhancing the proliferation and paracrine activity of adipose-derived stem cells. Stem Cells Dev. 29 (19), 1285–1293. 10.1089/scd.2020.0092 32703122

[B170] Okay E. T. Simsek C. Gunes A. Duruksu G. Gurbuz Y. (2015). Cross effects of resveratrol and mesenchymal stem cells on liver regeneration and homing in partially hepatectomized rats[J]. Stem Cell Rev. Rep. 11 (2), 322–31. 10.1007/s12015-014-9572-6 25416627

[B99] Pacyga K. Pacyga P. Topola E. Viscardi S. Duda-Madej A. (2024). Bioactive compounds from plant origin as natural antimicrobial agents for the treatment of wound infections. Int. J. Mol. Sci. 25 (4), 2100. 10.3390/ijms25042100 38396777 PMC10889580

[B100] Pang X. Zhong Z. Jiang F. Yang J. Nie H. (2022). Juglans regia L. extract promotes osteogenesis of human bone marrow mesenchymal stem cells through BMP2/Smad/Runx2 and Wnt/β-catenin pathways. J. Orthop. Surg. Res. 17 (1), 88. 10.1186/s13018-022-02949-1 35164786 PMC8842536

[B101] Pharoun J. Berro J. Sobh J. Abou-Younes M. M. Nasr L. Majed A. (2024). Mesenchymal stem cells biological and biotechnological advances: implications for clinical applications. Eur. J. Pharmacol. 977, 176719. 10.1016/j.ejphar.2024.176719 38849038

[B102] Pirmoradi S. Fathi E. Farahzadi R. Pilehvar-Soltanahmadi Y. Zarghami N. (2018). Curcumin affects adipose tissue-derived mesenchymal stem cell aging through TERT gene expression. Drug Res. (Stuttg) 68 (4), 213–221. 10.1055/s-0043-119635 29017189

[B103] Poon E.N.-Y. Luo X.-l. Webb S. E. Yan B. Zhao R. Wu S. C. M. (2020). The cell surface marker CD36 selectively identifies matured, mitochondria-rich hPSC-cardiomyocytes. Cell Res. 30 (7), 626–629. 10.1038/s41422-020-0292-y 32157205 PMC7343859

[B104] Preda M. B. Neculachi C. A. Fenyo I. M. Vacaru A. M. Publik M. A. Simionescu M. (2021). Short lifespan of syngeneic transplanted MSC is a consequence of *in vivo* apoptosis and immune cell recruitment in mice. Cell Death Dis. 12 (6), 566. 10.1038/s41419-021-03839-w 34075029 PMC8169682

[B105] Pu X. Chai Y. Guan L. Li W. Gao J. Jiang Z. (2020). Astragalus improve aging bone marrow mesenchymal stem cells (BMSCs) vitality and osteogenesis through VD-FGF23-Klotho axis. Int. J. Clin. Exp. Pathol. 13 (4), 721–729. 10.62347/IJCEP.2020.13.4.721 32355520 PMC7191145

[B106] Qin L. Wu X. Block M. L. Liu Y. Breese G. R. Hong J. S. (2007). Systemic LPS causes chronic neuroinflammation and progressive neurodegeneration. Glia 55 (5), 453–462. 10.1002/glia.20467 17203472 PMC2871685

[B107] Rahmani-Moghadam E. Talaei-Khozani T. Zarrin V. Vojdani Z. (2021). Thymoquinone loading into hydroxyapatite/alginate scaffolds accelerated the osteogenic differentiation of the mesenchymal stem cells. Biomed. Eng. Online 20 (1), 76. 10.1186/s12938-021-00916-1 34348708 PMC8336257

[B108] Rahmani-Moghadam E. Zarrin V. Mahmoodzadeh A. Owrang M. Talaei-Khozani T. (2022). Comparison of the characteristics of breast milk-derived stem cells with the stem cells derived from the other sources: a comparative review. Curr. Stem Cell Res. Ther. 17 (1), 71–90. 10.2174/1574888x16666210622125309 34161214

[B109] Rangasamy T. Ghimire L. Jin L. Le J. Periasamy S. Paudel S. (2021). Host defense against *Klebsiella pneumoniae* pneumonia is augmented by lung-derived mesenchymal stem cells. J. Immunol. 207 (4), 1112–1127. 10.4049/jimmunol.2000688 34341173 PMC8355100

[B110] Rendra E. Crigna A. T. Daniele C. Sticht C. Cueppers M. Kluth M. A. (2023). Clinical-grade human skin-derived ABCB5+ mesenchymal stromal cells exert anti-apoptotic and anti-inflammatory effects *in vitro* and modulate mRNA expression in a cisplatin-induced kidney injury murine model. Front. Immunol. 14, 1228928. 10.3389/fimmu.2023.1228928 38274791 PMC10808769

[B111] Rodríguez-Eguren A. Gómez-Álvarez M. Francés-Herrero E. Romeu M. Ferrero H. Seli E. (2022). Human umbilical cord-based therapeutics: stem cells and blood derivatives for female reproductive. medicine 23 (24), 15942 10.3390/ijms232415942 PMC9785531 36555583

[B112] Samsonraj R. M. Raghunath M. Nurcombe V. Hui J. H. van Wijnen A. J. Cool S. M. (2017). Concise review: multifaceted characterization of human mesenchymal stem cells for use in regenerative medicine. Stem Cells Transl. Med. 6 (12), 2173–2185. 10.1002/sctm.17-0129 29076267 PMC5702523

[B113] Santra M. Geary M. L. Rubin E. Hsu M. Y. S. Funderburgh M. L. Chandran C. (2024). Good manufacturing practice production of human corneal limbus-derived stromal stem cells and *in vitro* quality screening for therapeutic inhibition of corneal scarring. Stem Cell Res. Ther. 15 (1), 11. 10.1186/s13287-023-03626-8 38185673 PMC10773078

[B114] Seo Y. Shin T. H. Kim H. S. (2019). Current strategies to enhance adipose stem cell function: an update. Int. J. Mol. Sci. 20 (15), 3827. 10.3390/ijms20153827 31387282 PMC6696067

[B115] Serteyn D. Storms N. Mouithys-Mickalad A. Sandersen C. Niesten A. Duysens J. (2024). Revealing the therapeutic potential of muscle-derived mesenchymal stem/stromal cells: an *in vitro* model for equine laminitis based on activated neutrophils, anoxia-reoxygenation, and myeloperoxidase. Anim. (Basel) 14 (18), 2681. 10.3390/ani14182681 PMC11428732 39335269

[B116] Seyedi Z. Amiri M. S. Mohammadzadeh V. Hashemzadeh A. Haddad-Mashadrizeh A. Mashreghi M. (2023). Icariin: a promising natural product in biomedicine and tissue engineering. J. Funct. Biomater. 14 (1), 44. 10.3390/jfb14010044 36662090 PMC9862744

[B117] Shan Y. Zhang M. Tao E. Wang J. Wei N. Lu Y. (2024). Pharmacokinetic characteristics of mesenchymal stem cells in translational challenges. Signal Transduct. Target Ther. 9 (1), 242. 10.1038/s41392-024-01936-8 39271680 PMC11399464

[B118] Sharifi S. Moghaddam F. A. Abedi A. Maleki Dizaj S. Ahmadian S. Abdolahinia E. D. (2020). Phytochemicals impact on osteogenic differentiation of mesenchymal stem cells. Biofactors 46 (6), 874–893. 10.1002/biof.1682 33037744

[B119] Sheng H. Rui X. F. Sheng C. J. Li W. J. Cheng X. Y. Jhummon N. P. (2013). A novel semisynthetic molecule icaritin stimulates osteogenic differentiation and inhibits adipogenesis of mesenchymal stem cells. Int. J. Med. Sci. 10 (6), 782–789. 10.7150/ijms.6084 23630444 PMC3638303

[B120] Si Y. C. Li Q. Xie C. E. Niu X. Xia X. H. Yu C. Y. (2014). Chinese herbs and their active ingredients for activating xue (blood) promote the proliferation and differentiation of neural stem cells and mesenchymal stem cells. Chin. Med. 9 (1), 13. 10.1186/1749-8546-9-13 24716802 PMC3991899

[B121] Song H. Zhang R. Liu Y. Wu J. Fan W. Wu J. (2024). Menstrual blood-derived endometrial stem cells ameliorate ovarian senescence by relieving oxidative stress-induced inflammation. Reprod. Sci. 10.1007/s43032-024-01739-w 39500850

[B122] Song L. H. Pan W. Yu Y. H. Quarles L. D. Zhou H. H. Xiao Z. S. (2006). Resveratrol prevents CsA inhibition of proliferation and osteoblastic differentiation of mouse bone marrow-derived mesenchymal stem cells through an ER/NO/cGMP pathway. Toxicol In Vitro 20 (6), 915–922. 10.1016/j.tiv.2006.01.016 16524694

[B123] Sun K. Fan M. (2025). Effects of BMSC-exosomes on the proliferation and migration of chondrocytes. J. Med. Biol. Eng. 45, 47–54. 10.1007/s40846-025-00926-7

[B124] Tayebi B. Babaahmadi M. Pakzad M. Hajinasrollah M. Mostafaei F. Jahangiri S. (2022). Standard toxicity study of clinical-grade allogeneic human bone marrow-derived clonal mesenchymal stromal cells. Stem Cell Res. Ther. 13 (1), 213. 10.1186/s13287-022-02899-9 35619148 PMC9137136

[B125] Thiruvenkataramani R. P. Abdul-Hafez A. Kesaraju T. Mohamed H. Ibrahim S. A. Othman A. (2024). Small extracellular vesicles derived from cord blood plasma and placental mesenchymal stem cells attenuate acute lung injury induced by lipopolysaccharide (LPS). Int. J. Mol. Sci. 26 (1), 75. 10.3390/ijms26010075 39795932 PMC11719573

[B126] Tian Z. Yu T. Liu J. Wang T. Higuchi A. (2023). Introduction to stem cells. Prog. Mol. Biol. Transl. Sci. 199, 3–32. 10.1016/bs.pmbts.2023.02.012 37678976

[B127] Trivanović D. Jauković A. Popović B. Krstić J. Mojsilović S. Okić-Djordjević I. (2015). Mesenchymal stem cells of different origin: comparative evaluation of proliferative capacity, telomere length and pluripotency marker expression. Life Sci. 141, 61–73. 10.1016/j.lfs.2015.09.019 26408916

[B169] Tseng P. C. Hou S. M. Chen R. J. Peng H. W. Hsieh C. F. Kuo M. L. (2011). Resveratrol promotes osteogenesis of human mesenchymal stem cells by upregulating RUNX2 gene expression via the SIRT1/FOXO3A axis[J]. J Bone Miner. Res. 26 (10), 2552–63. 10.1002/jbmr.460 21713995

[B128] Ullah M. Liu D. D. Thakor A. S. (2019). Mesenchymal stromal cell homing: mechanisms and strategies for improvement. iScience 15, 421–438. 10.1016/j.isci.2019.05.004 31121468 PMC6529790

[B129] Valipour B. Simorgh S. Mirsalehi M. Moradi S. Taghizadeh-Hesary F. Seidkhani E. (2024). Improvement of spatial learning and memory deficits by intranasal administration of human olfactory ecto-mesenchymal stem cells in an Alzheimer's disease rat model. Brain Res. 1828, 148764. 10.1016/j.brainres.2024.148764 38242524

[B130] Vauzour D. (2012). Dietary polyphenols as modulators of brain functions: biological actions and molecular mechanisms underpinning their beneficial effects. Oxid. Med. Cell Longev. 2012, 1–16. 10.1155/2012/914273 PMC3372091 22701758

[B131] Vizoso F. J. Eiro N. Cid S. Schneider J. Perez-Fernandez R. (2017). Mesenchymal stem cell secretome: toward cell-free therapeutic strategies in regenerative medicine. Int. J. Mol. Sci. 18 (9), 1852. 10.3390/ijms18091852 28841158 PMC5618501

[B132] Wang M. Huang W. Huang J. Shi J. Huang N. Luo Y. J. (2024). Traditional Chinese medicine in Alzheimer's disease: from the perspective of GSK-3β and tau hyperphosphorylation. 13, 100543. 10.1016/j.prmcm.2024.100543

[B133] Wang N. Y. Lu C. J. Chen X. H. (2005). Study on effect of ginsenoside Rg1 in promoting myocardiac vascular endothelial cell regeneration through induction on bone marrow stem cell's migration and differentiation in rabbits of myocardial infarction. Zhongguo Zhong Xi Yi Jie He Za Zhi 25 (10), 916–919. 16313117

[B134] Wang W. Shen Z. Tang Y. Chen B. Chen J. Hou J. (2022). Astragaloside IV promotes the angiogenic capacity of adipose-derived mesenchymal stem cells in a hindlimb ischemia model by FAK phosphorylation via CXCR2. Phytomedicine 96, 153908. 10.1016/j.phymed.2021.153908 35026516

[B135] Wang X. Ma S. Meng N. Yao N. Zhang K. Li Q. (2016). Resveratrol exerts dosage-dependent effects on the self-renewal and neural differentiation of hUC-MSCs. Mol. Cells 39 (5), 418–425. 10.14348/molcells.2016.2345 27109421 PMC4870190

[B167] Wang C. C. Wang C. H. Chen H. C. Cherng J. H. Chang S. J. Wang, Y. W. (2018). Combination of resveratrol-containing collagen with adipose stem cells for craniofacial tissue-engineering applications[J]. Int. Wound. J. 15 (4), 660–672. 10.1111/iwj.12910 29536622 PMC7949979

[B136] Wang Y. Wang S. Zhang K. Zhang Z. Wan D. Guo X. (2019). Current situation of injury and its influencing factors among pupils and middle school students in Yanqing district, Beijing, 2017. Pract. Prev. Med. (10), 4. 10.3969/j.issn.1006-3110.2019.10.011

[B137] Wang Z. Jiang R. Wang L. Chen X. Xiang Y. Chen L. (2020). Ginsenoside Rg1 improves differentiation by inhibiting senescence of human bone marrow mesenchymal stem cell via GSK-3β and β-catenin. Stem Cells Int. 2020, 1–16. 10.1155/2020/2365814 PMC7271209 32565825

[B138] Wang Z. Wang L. Jiang R. Li C. Chen X. Xiao H. (2021). Ginsenoside Rg1 prevents bone marrow mesenchymal stem cell senescence via NRF2 and PI3K/Akt signaling. Free Radic. Biol. Med. 174, 182–194. 10.1016/j.freeradbiomed.2021.08.007 34364981

[B139] Wilkinson A. C. Igarashi K. J. Nakauchi H. (2020). Haematopoietic stem cell self-renewal *in vivo* and *ex vivo* . Nat. Rev. Genet. 21 (9), 541–554. 10.1038/s41576-020-0241-0 32467607 PMC7894993

[B140] Wu J. Chen J. Ge Y. Huang N. Luo Y. (2024). Neuroprotective effect of tanshinone IIA-modified mesenchymal stem cells in a lipopolysaccharide-induced neuroinflammation model. Heliyon 10 (8), e29424. 10.1016/j.heliyon.2024.e29424 38638958 PMC11024610

[B141] Wu J. J. Yang Y. Wan Y. Xia J. Xu J. F. Zhang L. (2022). New insights into the role and mechanisms of ginsenoside Rg1 in the management of Alzheimer's disease. Biomed. Pharmacother. 152, 113207. 10.1016/j.biopha.2022.113207 35667236

[B142] Wu T. Shu T. Kang L. Wu J. Xing J. Lu Z. (2017). Icaritin, a novel plant-derived osteoinductive agent, enhances the osteogenic differentiation of human bone marrow- and human adipose tissue-derived mesenchymal stem cells. Int. J. Mol. Med. 39 (4), 984–992. 10.3892/ijmm.2017.2906 28260001

[B143] Xie J. Wang H. Song T. Wang Z. Li F. Ma J. (2013). Tanshinone IIA and astragaloside IV promote the migration of mesenchymal stem cells by up-regulation of CXCR4. Protoplasma 250 (2), 521–530. 10.1007/s00709-012-0435-1 22872094

[B144] Xie L. Zhu Q. Lu J. J. C. (2022). Can we use Ginkgo biloba extract to treat Alzheimer’s disease? Lessons from preclinical and clinical studies. Cells 11 (3), 479. 10.3390/cells11030479 35159288 PMC8833923

[B145] Yang A. Yu C. Lu Q. Li H. Li Z. He C. (2019). Mechanism of action of icariin in bone marrow mesenchymal stem cells. Stem Cells Int. 2019, 1–12. 10.1155/2019/5747298 PMC6476003 31089330

[B146] Yang Y. Jiang C. Zeng J. Guo X. Chen M. Wu B. (2025). hsa_circ_0001599 promotes odontogenic differentiation of human dental pulp stem cells by increasing ITGA2 expression and stability. Commun. Biol. 8 (1), 74. 10.1038/s42003-025-07488-z 39825107 PMC11742660

[B147] Yang Y.-H. K. Ogando C. R. Wang See C. Chang T.-Y. Barabino G. A. (2018). Changes in phenotype and differentiation potential of human mesenchymal stem cells aging *in vitro* . Stem Cell Res. Ther. 9 (1), 131. 10.1186/s13287-018-0876-3 29751774 PMC5948736

[B148] Yaylacı S. Kaçaroğlu D. Hürkal Ö. Ulaşlı A. M. (2023). An enzyme-free technique enables the isolation of a large number of adipose-derived stem cells at the bedside. Sci. Rep. 13 (1), 8005. 10.1038/s41598-023-34915-0 37198228 PMC10192379

[B149] Ye W. Liu Z. Liu Y. Xiao H. Tan Q. Yan A. (2024). METTL3 promotes the osteogenic differentiation of periosteum-derived MSCs via regulation of the HOXD8/ITGA5 axis in congenital pseudarthrosis. Regen. Ther. 26, 42–49. 10.1016/j.reth.2024.04.004 38818480 PMC11137358

[B150] Yoon D. S. Choi Y. Choi S. M. Park K. H. Lee J. W. (2015). Different effects of resveratrol on early and late passage mesenchymal stem cells through β-catenin regulation. Biochem. Biophys. Res. Commun. 467 (4), 1026–1032. 10.1016/j.bbrc.2015.10.017 26456654

[B151] Young T. Z. Liu P. Urbonaite G. Acar M. (2019). Quantitative insights into age-associated DNA-repair inefficiency in single cells. Cell Rep. 28 (8), 2220–2230.e7. 10.1016/j.celrep.2019.07.082 31433994 PMC6744837

[B152] Yu S. Yu S. Liu H. Liao N. Liu X. (2023). Enhancing mesenchymal stem cell survival and homing capability to improve cell engraftment efficacy for liver diseases. Stem Cell Res. Ther. 14 (1), 235. 10.1186/s13287-023-03476-4 37667383 PMC10478247

[B153] Yu W. Lv Y. Xuan R. Han P. Xu H. Ma X. (2024). Human placental mesenchymal stem cells transplantation repairs the alveolar epithelial barrier to alleviate lipopolysaccharides-induced acute lung injury. Biochem. Pharmacol. 229, 116547. 10.1016/j.bcp.2024.116547 39306309

[B168] Yuan P. Qin H. Y. Wei J.Y. Chen G. Li X. (2022). Proteomics reveals the potential mechanism of Tanshinone IIA in promoting the Ex Vivo expansion of human bone marrow mesenchymal stem cells[J]. Regen Ther. 21, 560–573. 10.1016/j.reth.2022.11.004 36475023 PMC9700269

[B154] Yun W. S. Cho H. Jeon S. I. Lim D.-K. Kim K. J. B. (2023). Fluorescence-based mono- and multimodal imaging for *in vivo* tracking of mesenchymal stem cells. Stem Cells 13 (12), 1787. 10.3390/biom13121787 PMC10742164 38136656

[B155] Yun Y. C. Jeong S. G. Kim S. H. Cho G. W. (2019). Reduced sirtuin 1/adenosine monophosphate-activated protein kinase in amyotrophic lateral sclerosis patient-derived mesenchymal stem cells can be restored by resveratrol. J. Tissue Eng. Regen. Med. 13 (1), 110–115. 10.1002/term.2776 30479062

[B156] Zeng N. Chen H. Wu Y. Liu Z. (2022). Adipose stem cell-based treatments for wound healing, Front. Cell Dev. Biol., 9. 821652. 10.3389/fcell.2021.821652 35087840 PMC8787271

[B157] Zeng X. Zheng Y. Liu Y. Su W. (2021). Chemical composition, quality control, pharmacokinetics, pharmacological properties and clinical applications of Fufang Danshen Tablet: a systematic review. J. Ethnopharmacol. 278, 114310. 10.1016/j.jep.2021.114310 34107328

[B158] Zhang J. Wu C. Gao L. Du G. Qin X. (2020). Astragaloside IV derived from Astragalus membranaceus: a research review on the pharmacological effects. Adv. Pharmacol. 87, 89–112. 10.1016/bs.apha.2019.08.002 32089240

[B159] Zhang P. Dong J. Fan X. Yong J. Yang M. Liu Y. (2023). Characterization of mesenchymal stem cells in human fetal bone marrow by single-cell transcriptomic and functional analysis. Signal Transduct. Target Ther. 8 (1), 126. 10.1038/s41392-023-01338-2 36997513 PMC10063684

[B160] Zhang X.-b. Chen X.-y. Qi J. Zhou H.-y. Zhao X.-b. Hu Y.-c. (2022). New hope for intervertebral disc degeneration: bone marrow MesenchymalStem cells and exosomes derived from bone marrow MesenchymalStem cell transplantation. stem cell Transplant. 22 (4), 291–302. 10.2174/1566523221666211012092855 34636308

[B161] Zhang X. M. Ma J. Sun Y. Yu B. Q. Jiao Z. M. Wang D. (2018). Tanshinone IIA promotes the differentiation of bone marrow mesenchymal stem cells into neuronal-like cells in a spinal cord injury model. J. Transl. Med. 16 (1), 193. 10.1186/s12967-018-1571-y 30001730 PMC6044071

[B162] Zhang Y. Ravikumar M. Ling L. Nurcombe V. Cool S. M. (2021). Age-related changes in the inflammatory status of human mesenchymal stem cells: implications for cell therapy. Stem Cell Rep. 16 (4), 694–707. 10.1016/j.stemcr.2021.01.021 PMC8072029 33636113

[B163] Zheng P. D. Mungur R. Zhou H. J. Hassan M. Jiang S. N. Zheng J. S. (2018). Ginkgolide B promotes the proliferation and differentiation of neural stem cells following cerebral ischemia/reperfusion injury, both *in vivo* and *in vitro* . Neural Regen. Res. 13 (7), 1204–1211. 10.4103/1673-5374.232476 30028328 PMC6065216

[B164] Zhidu S. Ying T. Rui J. Chao Z. (2024). Translational potential of mesenchymal stem cells in regenerative therapies for human diseases: challenges and opportunities. Stem Cell Res. Ther. 15 (1), 266. 10.1186/s13287-024-03885-z 39183341 PMC11346273

[B165] Zhou J. Shi Y. (2023). Mesenchymal stem/stromal cells (MSCs): origin, immune regulation, and clinical applications. Cell Mol. Immunol. 20 (6), 555–557. 10.1038/s41423-023-01034-9 37225837 PMC10229593

[B166] Zhou T. Yuan Z. Weng J. Pei D. Du X. He C. (2021). Challenges and advances in clinical applications of mesenchymal stromal cells. J. Hematol. Oncol. 14 (1), 24. 10.1186/s13045-021-01037-x 33579329 PMC7880217

